# Plasma EV Proteomics Identifies ECM Remodeling and Inflammatory Proteins LUM and C7 as Candidate Biomarkers in FSHD

**DOI:** 10.1002/acn3.70435

**Published:** 2026-05-20

**Authors:** Mustafa Bilal Bayazit, Chiranth K. Nagaraj, Jackson S. Newell, Kim Truc Nguyen, Xilal Y. Rima, Jacob Doon‐Ralls, Eduardo Reátegui, Jeffrey M. Statland, Rabi Tawil, Kevin M. Flanigan, Scott Q. Harper, Nizar Y. Saad

**Affiliations:** ^1^ Jerry R. Mendell Center for Gene Therapy Abigail Wexner Research Institute at Nationwide Children's Hospital Columbus Ohio USA; ^2^ William G. Lowrie Department of Chemical and Biomolecular Engineering The Ohio State University Columbus Ohio USA; ^3^ Diabetes and Metabolism Research Center The Ohio State University Wexner Medical Center Columbus Ohio USA; ^4^ Comprehensive Cancer Center The Ohio State University Columbus Ohio USA; ^5^ Department of Neurology University of Kansas Medical Center Kansas City KA USA; ^6^ Department of Neurology University of Rochester Medical Center Rochester New York USA; ^7^ Department of Pediatrics The Ohio State University College of Medicine Columbus Ohio USA

**Keywords:** Extracellular vesicles, Facioscapulohumeral muscular dystrophies, Protein biomarkers

## Abstract

**Objective:**

Facioscapulohumeral muscular dystrophy (FSHD) is one of the most debilitating and common muscular dystrophies. Despite its severity, no approved therapy exists for FSHD patients. However, several therapeutic candidates are currently under development, and some have recently entered clinical trials, marking the need for reliable biomarkers and outcome measures to monitor disease progression and treatment response in FSHD. To date, clinicians have relied primarily on validated functional and patient‐reported outcome measures, while circulating molecular biomarkers remain unvalidated.

**Methods:**

Here, we investigated the plasma extracellular vesicle (EV) proteome to identify protein biomarkers that could be more reliably and conveniently used for FSHD prognosis, patient stratification for clinical trials and treatment response. In this study, we searched for EV protein biomarkers using plasma from 45 FSHD1 patients and 28 healthy controls distributed across two independent cohorts.

**Results:**

Using integrated, batch‐corrected mass spectrometry analyses, we identified Lumican (LUM), along with several complement and extracellular matrix–associated proteins, as consistently upregulated EV proteins in FSHD. Additionally, LUM levels correlated with disease severity (FSHD‐COM score; sex‐adjusted Partial Pearson *R*: 0.454, *p*: 0.039) and other continuous clinical outcome measures (self‐selected gait speed time; Pearson *R*: −0.485, *p*: 0.022 and timed up and go; Pearson *R*: 0.463, *p*: 0.034).

**Interpretation:**

Our findings demonstrate that profiling circulating EV content in FSHD plasma can reveal EV protein biomarkers that warrant further validation in larger cohorts to assess their potential clinical relevance as minimally invasive surrogate molecular biomarkers for FSHD.

## 1 Introduction

Facioscapulohumeral muscular dystrophy (FSHD) is the third most common muscular dystrophy affecting approximately 1 in 8333 individuals worldwide [1, 2, 3]. It is characterized by progressive skeletal muscle weakness and wasting, particularly in the face, shoulders, upper arms, and lower extremities. FSHD is an autosomal dominant or digenic disorder caused by the de‐repression of the transcription factor DUX4 in skeletal muscle. DUX4 expression in myofibers causes oxidative stress, inflammation, and activation of a gene expression cascade ultimately leading to cell death [4, 5, 6, 7, 8, 9]. In skeletal muscle, DUX4 induces inflammation and mononuclear immune cell infiltration, edema, fat infiltration, fibrosis, and muscle atrophy [4, 10, 11]. Currently, no approved therapy exists for FSHD, although several candidates are in the preclinical stage, and some have recently entered clinical trials [12, 13, 14, 15, 16, 17].

With therapeutic development, there is an urgent need for reliable biomarkers and outcome measures to monitor disease progression and treatment response in FSHD. Many established clinical endpoints, such as gene expression, rely on invasive procedures, including muscle biopsies. In addition, DUX4 is unreliable as a biomarker due to its extremely low and heterogeneous expression in myonuclei [18, 19]. To date, no robust blood‐based biomarker has been validated for FSHD, with only subtle increases in plasma complement proteins reported in patients using targeted approaches [20, 21, 22]. So far, clinicians rely on functional and patient‐reported outcome measures to evaluate disease progression and therapeutic efficacy in FSHD clinical trials [23, 24, 25, 26]. Despite their rigorous validation, these clinical outcome measures may vary across sites and remain labor‐intensive, invasive, time‐consuming, inconvenient for some patients, and expensive, which could delay approval of therapies. We hypothesize that the unbiased search for minimally invasive circulating molecular biomarkers will allow the identification of biomarkers that reliably reflect muscle disease activity and objectively predict therapeutic efficacy.

Extracellular vesicles (EVs) are lipid bilayer–delimited particles released by most cell types and carry proteins, nucleic acids, and lipids reflective of their cellular origin [27, 28]. EV cargo can be enclosed within the vesicle lumen, embedded in the membrane, or associated with an external protein corona. EVs participate in intercellular communication and are readily detectable in biofluids [29]. Circulating plasma EVs are emerging as promising disease biomarkers, as they are often enriched for disease‐associated molecules compared with crude plasma [30]. Skeletal muscle is a significant contributor to the circulating EV pool, and EV cargo is known to be selectively packaged in response to cellular stress and pathology [31, 32, 33, 34]. Despite this, the circulating FSHD EV proteome has not yet been profiled.

In this exploratory study, we present the first unbiased proteomic profiling of plasma‐derived EVs in FSHD1 (major FSHD subtype), analyzing 45 patients and 28 healthy controls distributed across two independent cohorts. Using integrated, batch‐corrected mass spectrometry analyses, we identified Lumican (LUM), along with complement and extracellular matrix–associated proteins, as consistently upregulated EV proteins in FSHD1. Particularly, LUM levels correlated with disease severity and other continuous clinical outcome measures. Additionally, we compiled a composite panel of upregulated EV proteins that efficiently distinguishes most FSHD1 patients from healthy controls across cohorts, and stratified individuals based on disease severity. This cross‐cohort consistency highlights the potential of EV‐based biomarkers for diagnostic and prognostic applications in FSHD.

## 2 Materials and Methods

### 2.1 Study Cohorts

In Cohort 1, de‐identified FSHD1 and healthy control plasma samples were obtained from Dr. Rabi Tawil (University of Rochester, NY, USA). FSHD1 samples were part of the clinical trial readiness to solve barriers to drug development in FSHD (ReSolve) study (STUDY00140842) [26]. This cohort comprised 24 genetically confirmed FSHD1 patients and 11 randomly identified healthy control volunteers, aged 19–72 years. For this cohort, 14 different clinical outcome measures were available for FSHD1 patients including the FSHD composite score (FSHD‐COM) (Table 1). This score is a comprehensive, 18‐item performance‐based functional composite outcome measure, assessing the legs, shoulders, and arms, trunk, hands, and balance/mobility [26]. Subjects were excluded from the study if they had cardiac or respiratory dysfunction, orthopedic conditions preventing safe muscle function testing, were pregnant, regularly used anabolic or catabolic agents, or had used an experimental drug in an FSHD clinical trial within the past 30 days. The plasma samples for Cohort 1 were collected between 2018–2021. Healthy control samples were collected from volunteers at the University of Rochester Medical center under a separate local IRB‐approved protocol (STUDY00000828). In Cohort 2, plasma samples were obtained from the “FSHD Lab Day” organized at Nationwide Children's Hospital in July 2023 under an IRB‐approved protocol (IRB13‐00190). This cohort included 17 genetically or clinically confirmed FSHD1 patients and 21 randomly identified asymptomatic healthy controls who were either relatives or non‐relatives of FSHD subjects. Cohort 2 subjects were 8–79 years old. For all samples, plasma was collected using anti‐coagulant (EDTA) treated collection tubes, and each sample was assigned a blinding code so that all batches of samples were run by the Saad lab in a blinded fashion (Table 1). Patient recruitment was performed using documents and protocols approved by the Human Subjects Independent Review Board (IRB) at the University of Rochester, Rochester, NY. and Nationwide Children's Hospital, Columbus, OH. All samples were de‐identified before analysis. Written informed consent was obtained from all subjects participating in the study. For minors, parental or legal guardian consent and participant assent, when appropriate, were obtained prior to enrollment.

**TABLE 1:** Demographics and clinical data of the two cohorts. CSS: Clinical Severity Score. FSHD‐COM: FSHD composite score.

	Cohort 1		Cohort 2	
	Healthy	FSHD1	Healthy	FSHD1
Age				
8–18	—	—	1	4
19–40	5	6	4	3
41–60	3	12	7	6
61–79	3	6	5	8
Sex				
F	8	11	10	12
M	3	13	7	9
CSS				
1–3	—	8	—	N.A.
4–6	—	8	—	N.A.
7–9	—	8		N.A.
FSHD‐COM				
1–15	—	9		N.A.
16–30	—	8		N.A.
31+	—	7		N.A.
Total #	**11**	**24**	**17**	**21**

### 2.2 Plasma Collection and EV Isolation

Whole blood samples were obtained in EDTA‐containing tubes (purple top) and stored on wet ice and centrifuged within 1 h. EVs were isolated from pre‐cleared plasma (500 μL) by size‐exclusion chromatography (SEC), using Izon qEV columns (35 nm), and following the most recent MISEV guidelines from the International Society for Extracellular Vesicles for isolation, storage, and characterizations [35]. The first four SEC fractions were pooled and concentrated using Amicon ultra‐4 centrifugal filter with 100 kDa MWCO (Millipore). EV size and concentration were monitored by Nanoparticle Tracking Analysis (NTA) using a Nanosight instrument (NS300, Malvern Panalytical, UK). Briefly, samples were diluted in sterile, filtered PBS to reach optimal particle concentration (1 × 10^7^–1 × 10^8^ particles/mL) and analyzed at room temperature. Two 30‐s videos were recorded with the camera level set at 13 and detection threshold of 3. Videos were processed using NTA software (version 3.2). EV structure was further characterized by transmission electron microscopy (TEM). EV quality was assessed by Western blot to evaluate the presence of plasma EV markers (e.g., CD9, flotillin and TSG101), and absence of cell markers (e.g., calnexin). EV isolation was performed blinded. Samples were then batched to ensure patient and healthy controls samples were analyzed together.

### 2.3 Protein Isolation and Mass Spectrometry

Mass spectrometry (MS) was conducted and performed blinded at The Ohio State University Proteomic Core. Plasma EV samples were subjected to trypsin digestion using the S‐trap approach and following the Core's protocol. A total of 100 μg of protein was used for MS. Liquid Chromatography was then performed with tandem MS (LC–MS/MS). Using Mascot Daemon by Matrix Science version 2.7.0 (Boston, MA) via Proteome Discoverer (version 2.4 Thermo Scientific), our data was searched against the most recent Uniprot databases. Label‐free quantitation was performed using the spectral count approach, in which the relative protein quantitation is measured by comparing the number of MS/MS spectra identified from the same protein in each of the multiple LC–MS/MS datasets.

### 2.4 Biostatistical Analysis

Principal component analysis (PCA) was conducted using *prcomp* (R) on variance‐stabilized or log‐normalized spectral count data. When applicable, ComBat‐corrected matrices were used to harmonize proteomic abundance data across cohorts processed under comparable pre‐analytical conditions, enabling integrated analyses: age and sex were modeled as covariates in downstream statistical analyses, and disease‐associated trends were also evaluated within individual cohorts prior to correction. PCA was used to visualize sample clustering, evaluate cohort effects, and confirm effective batch correction.

Partial Least Squares‐Discriminant Analysis (PLS‐DA) was performed using the *mixOmics* package (R) on log‐transformed, mean‐centered protein expression values to identify multivariate signatures distinguishing FSHD from healthy controls [36]. The optimal number of components was determined by cross‐validation. Variable Importance in Projection (VIP) scores were extracted from the final model, and proteins with the highest VIP scores were considered key contributors to class separation. To assess model robustness and reduce the risk of overfitting, repeated 5‐fold cross‐validation (100 repeats) and permutation testing (1000 permutations) were performed for the combined cohort and for each cohort separately. Overall error rate at component 2 was used as the primary performance metric, and permutation *p*‐values were calculated as the proportion of permuted models with equal or lower error rates than the observed model.

To adjust for cohort‐specific processing effects, ComBat (R package: *sva*) was applied to log‐transformed protein expression matrices. Cohort was modeled as the batch variable, while disease status was preserved [37]. The batch‐corrected matrix was used for PCA, differential expression, and composite *z*‐score analyses.

Protein‐level *z*‐scores, i.e., how many standard deviations the count of the biomarker is away from the mean count across all samples, were computed across samples using standard scaling. Composite *z*‐scores for each sample were generated by averaging *z*‐scores across a significant upregulated protein. These values summarize coordinated changes across a biomarker panel and were used in waterfall plots and correlation analyses.

Differential abundance of EV‐associated protein levels was assessed using the Wald test with a significance cut‐off of *p* < 0.05 within the DESeq2 package in *R* [38]. A total of 317 commonly detected proteins across both cohorts were included in the analysis. Low‐abundance proteins were removed using a detection filter requiring mean normalized counts ≥ 2 and ≥ 3 counts in≥one‐third of samples in at least one group. To control for testing of many proteins, false discovery rates (FDR) were computed from raw *p* values, with FDR < 0.1 and log2FC > 0.5 used as a cutoff for significance. Unless indicated, all DESeq2 analyses were performed using age and sex as covariates.

Pearson correlation coefficients with *p*‐values were calculated on scatter plots using the stat_cor function in R. Partial Pearson correlations adjusting for covariates were performed with the ppcor package [39]. Specifically, correlations between proteins and clinical outcome measures were adjusted for age, sex, or both, depending on each outcome measure's own association with those covariates.

Elastic‐net classification models were trained using *glmnet*, which applies combined L1/L2 regularization suitable for high‐dimensional correlated features [40]. Hyperparameters (*α*, *λ*) were tuned via repeated 10‐fold cross‐validation. Model performance was evaluated using Area Under the Curve (AUC), sensitivity, and specificity on held‐out test sets. Non‐zero coefficients in the final model represent biomarkers contributing to discrimination.

Specifically, the dataset was first split into training (75%) and hold‐out (25%) sets within Cohort 2. Elastic‐net model tuning (α and λ) was performed using cross‐validation on the training set only, and feature selection was based on the resulting penalized model coefficients. A compact generalized linear model was then refit using the selected features and evaluated on both the hold‐out set and the independent Cohort 1 dataset. To further evaluate model stability, permutation testing (1000 permutations) was performed by randomly shuffling training labels while preserving the hold‐out and external validation sets, followed by repetition of the full modeling pipeline. Permutation *p*‐values were calculated as the proportion of permuted models achieving AUC values equal to or greater than the observed model. Receiver operating characteristic (ROC) analyses were additionally performed using the pROC package for Lumican (LUM) alone and for the composite biomarker panel (LUM, C7, C9, and FBLN1).

### 2.5 Immunoblotting

50 μg of plasma EV proteins were used for Western blot validation. To verify the association of the biomarkers to EVs, 100 μg of EV proteins were treated with Proteinase K (20 μg/mL) for 1 h at 37°C, to only retain the proteins packaged within the EVs and protected by their lipid bilayer membrane. PMSF (20 μM) was added to the EVs for 10 min at room temperature (RT) post treatment to timely quench Proteinase K activity. As an additional control, EV membranes were treated simultaneously with Proteinase K and 1% Triton X‐100, which ruptures EV membrane, allowing Proteinase K to digest intraluminal EV proteins.

To verify the specificity of the LUM antibody, LUM was deglycosylated using PNGase F Glycan Cleavage Kit (A39245, Gibco). Briefly, 25 μg of EV proteins were incubated with either PBS or recombinant PNGase F mix at 50°C for one hour. EVs were then boiled in Laemmli buffer, and 50 μg of proteins were size‐separated (25 μg for PNGase F experiments) using 4%–20% TGX Stain‐Free Protein Gels (Mini‐PROTEAN, BioRad). Total protein levels were observed using stain‐free imaging (ChemiDoc, BioRad). Primary antibodies used in this study were purchased from Proteintech: rabbit‐anti CD9 (20597–1‐AP, 1:1000–1:2000), rabbit‐anti C7 (17642–1‐AP, 1:1000), rabbit‐anti LUM (10677–1‐AP, 1:500), mouse‐anti flotillin (67968–1‐Ig, 1:5000), mouse‐anti calnexin (66903–1‐Ig, 1:5000), mouse‐anti‐TSG101 (MA1‐23296, 1:500), and rabbit‐anti ApoB (20578–1‐AP, 1:1500). Uncropped Western blot images relating to Figure 3B,E can be found in supplementary figures (Figure S4A,B).

### 2.6 Elisa

Plasma EV LUM levels were detected using the Human Lumican ELISA kit (ab315054, Abcam) and following the manufacturer's protocol. LUM levels were detected at 450 nm and were normalized with protein content.

### 2.7 Transmission Electron Microscopy (TEM) and Imaging

Nickel TEM grids (200‐mesh, lacy carbon film; Cat. No. 01808 N, Ted Pella Inc.) were placed on a clean parafilm surface. To functionalize the grids, 20 μL of anti‐rabbit IgG conjugated to 5 nm gold nanoparticles (Cat. No. G7277‐.4ML, Sigma‐Aldrich) was drop casted and incubated for 60 min at room temperature. The grids were washed twice with PBS to remove residual gold nanoparticles. Grids were subsequently blocked with 10 μL of 10% normal goat serum (Cat. No. 50062Z, Life Technologies Corp.) for 60 min to minimize nonspecific binding. EV samples were bio‐conjugated by incubation with Lumican rabbit polyclonal antibody (1 μg/mL; Cat. No. 10677–1‐AP, Proteintech) for 60 min in a 1% bovine serum albumin (Cat No. BP9703‐100, Thermo‐Fisher Inc.) solution. The antibody‐conjugated EVs were purified using SEC qEV columns (35 nm cutoff) according to the manufacturer's protocol, with four sequential fractions collected per sample. For TEM analysis, 10 μL of Lumican antibody–conjugated EVs were drop‐cast onto individual TEM grids and incubated for 60 min. The grids were washed twice with PBS to remove residual Lumican antibody‐conjugated EVs. Excess liquid was blotted, and grids were negatively stained with 20 μL of UranyLess EM contrast stain (Electron Microscopy Sciences) for 22 s. Staining was followed by two sequential washes with 20 μL of DI water. Grids were then placed in a grid storage box and dried overnight in the dark at ambient conditions. TEM imaging was performed using a Tecnai TF‐20 microscope (Thermo‐Fisher Inc.; Center for Electron Microscopy and Analysis, OH, USA) operated at 300 kV. Quantification of immunogold TEM images to determine the percentage of LUM‐positive EVs and mean number of gold nanoparticles (AuNPs) bound per EV vesicle was done using ImageJ (Fiji).

## 3 Results

### 3.1 Disease‐Specific Clustering Highlights Inflammation and ECM Signatures in Plasma EV Proteomes

**FIGURE 1:**
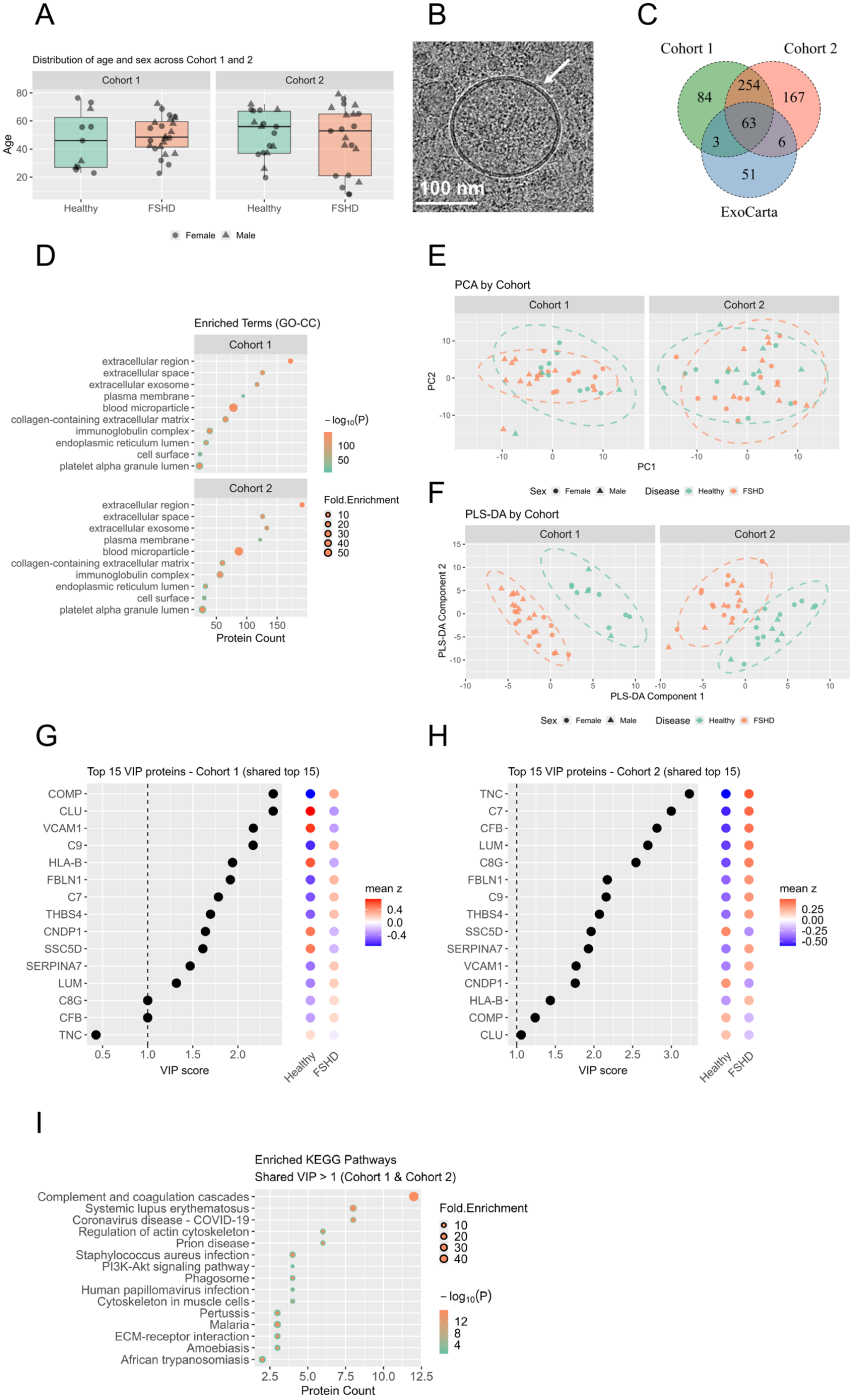
Characterization of study subjects and their isolated plasma EVs and EV proteomes. (A) Distribution of age and sex across Cohort 1 and 2. (B) EVs visualized with Transmission Electron Microscopy. Lipid bilayer of EV indicated with white arrow. (C) Composite profiles of 12 plasma EV samples (7 healthy and 5 FSHD1) assessed by Nanoparticle Tracking Analysis, displaying average EV size and concentration. (D) Venn diagram of overlapping proteins between this study and the ExoCarta Database for plasma EV proteins. (E) Enrichment analysis of proteins identified in this study. This analysis was conducted with DAVID software for Gene Ontology (GO) Terms with Cellular Compartment sub‐analysis. Top ten significantly (Bonferroni corrected *p* < 0.05) enriched terms are displayed. (F) Principal component analysis (PCA) of proteins identified by mass spectrometry. Dashed ellipses represent the 95% confidence region of samples from a given disease state (FSHD1 or Healthy), based on their PC1/PC2 positions. (G) Supervised partial least squares‐discriminant analysis (PLS‐DA) of plasma EV proteome. PLS‐DA components of cohorts display variance between disease groups. Confidence intervals (95%) are indicated by ellipses. (H,I) Variable Importance in Projection (VIP) scores displaying 15 proteins that commonly contribute to the variance between the groups across cohorts. Variation is caused by proteins with VIP > 1. Key heatmaps of relative protein abundance displayed as *z*‐scores are also illustrated. (J) Enrichment analysis of proteins that commonly contribute to the variance between the groups across cohorts in this study. This analysis was conducted with DAVID software for KEGG pathway. Top 15 significantly (Bonferroni corrected *p* < 0.05) enriched pathways are displayed.

We analyzed plasma EVs from two independent cross‐sectional FSHD1 cohorts (Table 1 and Figure 1A). We isolated plasma EVs using size‐exclusion chromatography and characterized them by transmission electron microscopy (TEM), which confirmed their typical round morphology and delimitation by a lipid bilayer membrane (Figure 1B).

To determine the EV proteome, we performed LC–MS/MS analysis, which enables sensitive, unbiased profiling of EV‐associated proteins. In total, we identified 404 proteins in Cohort 1 and 490 proteins in Cohort 2, with 317 proteins shared across both cohorts (Table S1). Of these, 63 proteins were listed in the ExoCarta plasma EV database (http://exocarta.org, accessed July 23, 2024) (Figure 1C). Gene Ontology (GO) enrichment analysis showed that the identified proteins were enriched in terms related to the extracellular region, extracellular exosome, and extracellular space [41] (Figure 1D).

We next performed principal component analysis (PCA) to assess the overall EV protein profile clusters. No separation in global proteome between FSHD1 patients and healthy controls was observed in PCA plots of either cohort (Figure 1E). To determine disease‐specific clustering, we next performed Partial Least Squares‐Discriminant Analysis (PLS‐DA), which is a supervised clustering analysis that incorporates all proteomic data while also explicitly weighing components that maximize separation between predefined classes (FSHD1 vs. Healthy). This is especially common in EV biomarker discovery where studies rely on PLS‐DA rather than PCA to reveal disease‐related structure in EVs [42]. PLS‐DA suggested separation between FSHD1 patients and healthy controls (Figure 1F). To assess model robustness, repeated cross‐validation and permutation testing (*n* = 1000) were performed for each cohort. Cohort 1 showed discriminatory performance (error rate = 0.29; approximately 71% accuracy) with statistically significant support (*p* = 0.045), whereas Cohort 2 showed limited discriminatory ability (error rate = 0.48; approximately 52% accuracy; *p* = 0.45), indicating that the supervised separation was not consistently reproducible across cohorts (Figure S1A,B).

Furthermore, PLS‐DA analysis allows identification of molecules that contribute to class separation by calculating Variable Importance in Projection (VIP) scores. The variation is caused by those with VIP scores > 1. By doing so, we consistently observed proteins of the complement activation pathway, inflammation, and extracellular matrix (ECM), which drove the separation between FSHD1 patients and healthy controls in both cohorts (Figure 1G–I and Table S2).

### 3.2 Cross‐Cohort Differential Proteomics Highlights Lumican (LUM) and Complement Component 7 (C7) Enrichment in FSHD1 Extracellular Vesicles

To investigate disease‐associated changes, we performed differential protein abundance analysis between FSHD1 patients and healthy controls in each cohort (Table S3). In Cohort 1 and 2, six and nine proteins were differentially abundant at a fold‐change of |log2FC| > 0.5 and nominal significance of *p* < 0.05, respectively (Tables S4 and S5). In Cohort 2, three proteins also met the significance criterion of |log2FC| > 0.5 and false discovery rate (FDR) < 0.1 (Figure S2A–D). Among these, Carnosine Dipeptidase 1 (CNDP1) was consistently downregulated in both cohorts (Cohort 1: log2FC = −1.035, *p* = 0.031; Cohort 2: log2FC = −1.482, *p* = 0.019) (Figure S2E). KEGG pathway analysis of differentially abundant proteins (*p* < 0.05) revealed that the top enriched pathway in both cohorts was the complement and coagulation cascades, suggesting consistent dysregulation of this pathway in FSHD1 patients (Figure S2F). Other enriched pathways in both cohorts are inflammation as well as ECM‐receptor interaction and regulation of actin cytoskeleton. The latter two pathways are directly connected to maintain a strong cellular structure and function [43]. The enrichment of these pathways indicates possible remodeling of the cell‐matrix interface due to disruption in the cell's physical architecture and its signaling response to inflammation.

**FIGURE 2:**
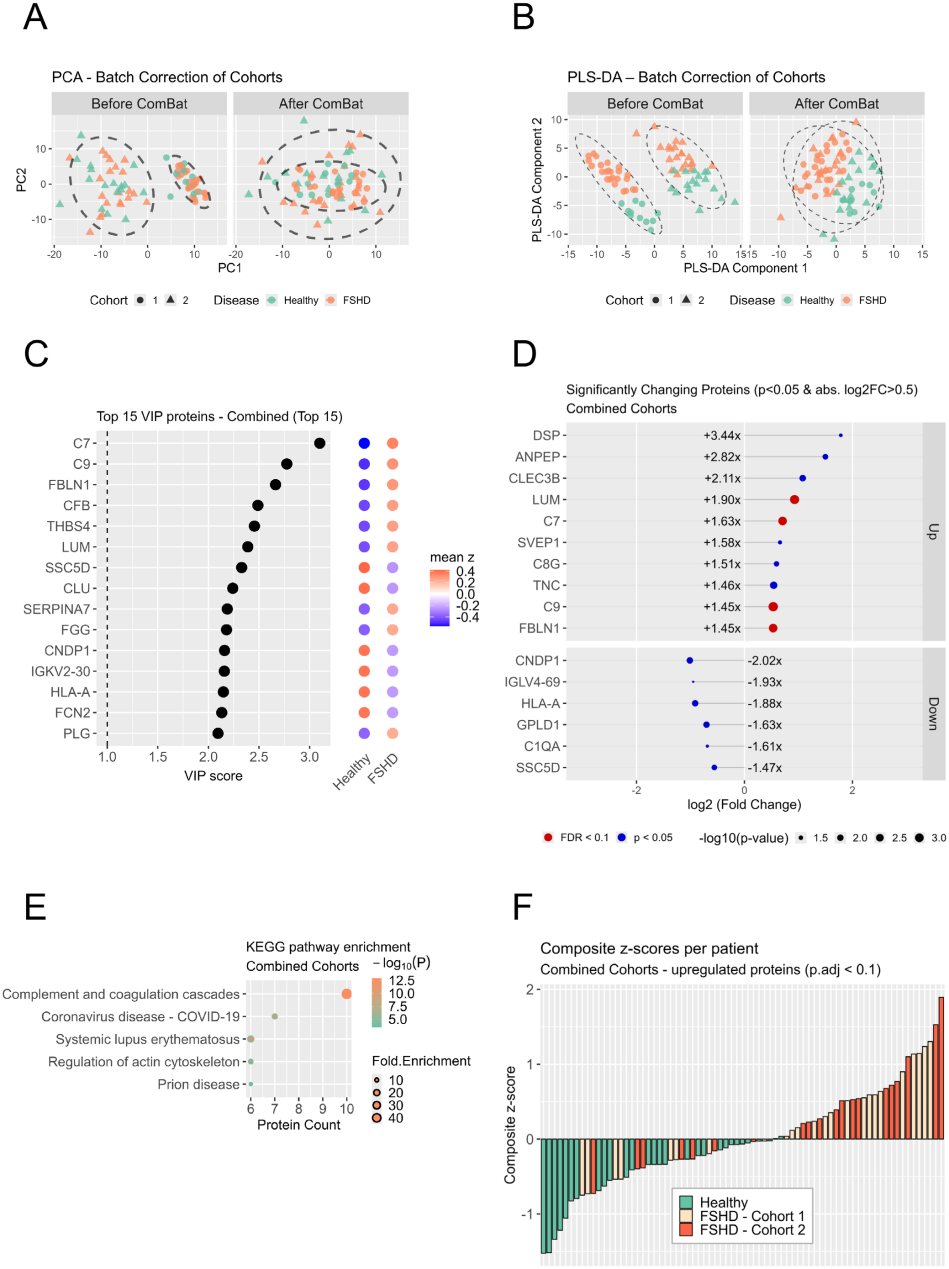
Combination of cohorts. (A) Batch specific clustering of cohorts, before and after ComBat batch correction. Dashed ellipse represents the 95% confidence region of samples from a given cohort, based on their PC1/PC2 positions. (B) Supervised partial least squares‐discriminant analysis (PLS‐DA) of uncorrected and batch‐corrected EV proteome. PLS‐DA components of cohorts display variance between cohorts with 95% confidence intervals indicated by ellipses. (C) Variable Importance in Projection (VIP) scores displaying 15 proteins that contribute to the variance between the groups across cohorts. Variation is caused by proteins with VIP > 1. Key heatmaps of relative protein abundance displayed as *z*‐scores are also illustrated. (D) Lollipop plot illustrating significantly changing proteins in the combined data. Absolute fold changes are labeled on the plot. *p* is calculated with Wald‐test. Differential abundance analyses were adjusted for age and sex (see also Table 2). (E) Enriched KEGG pathways (Bonferroni corrected *p* < 0.05) conducted with DAVID software. (F) A composite (mean) *z*‐score of five upregulated (*p* < 0.05) proteins was calculated. Waterfall plot illustrates ranking of each participant based on the composite *z*‐score.

Given the clinical heterogeneity of FSHD1, we next combined the datasets to increase statistical power. To mitigate cohort‐specific technical variation, we applied ComBat batch correction, an empirical Bayes method that removes non‐biological batch effects while preserving genuine biological differences. This homogenization of cohorts is illustrated in PCA‐plots before and after batch correction (Figure 2A). PLS‐DA plots confirmed that the disease‐specific differences were apparent after batch correction (Figure 2B). Validation of the combined PLS‐DA model demonstrated moderate classification performance after batch correction (error rate = 0.37; approximately 63% accuracy), and permutation testing confirmed that the observed separation exceeded random expectation (*p* = 0.026), supporting a disease‐associated multivariate structure after harmonization (Figure S3). Furthermore, proteins that separate FSHD1 and healthy controls (i.e., highest VIPs) were the ones identified in the single‐cohorts (Figure 2C).

**FIGURE 3:**
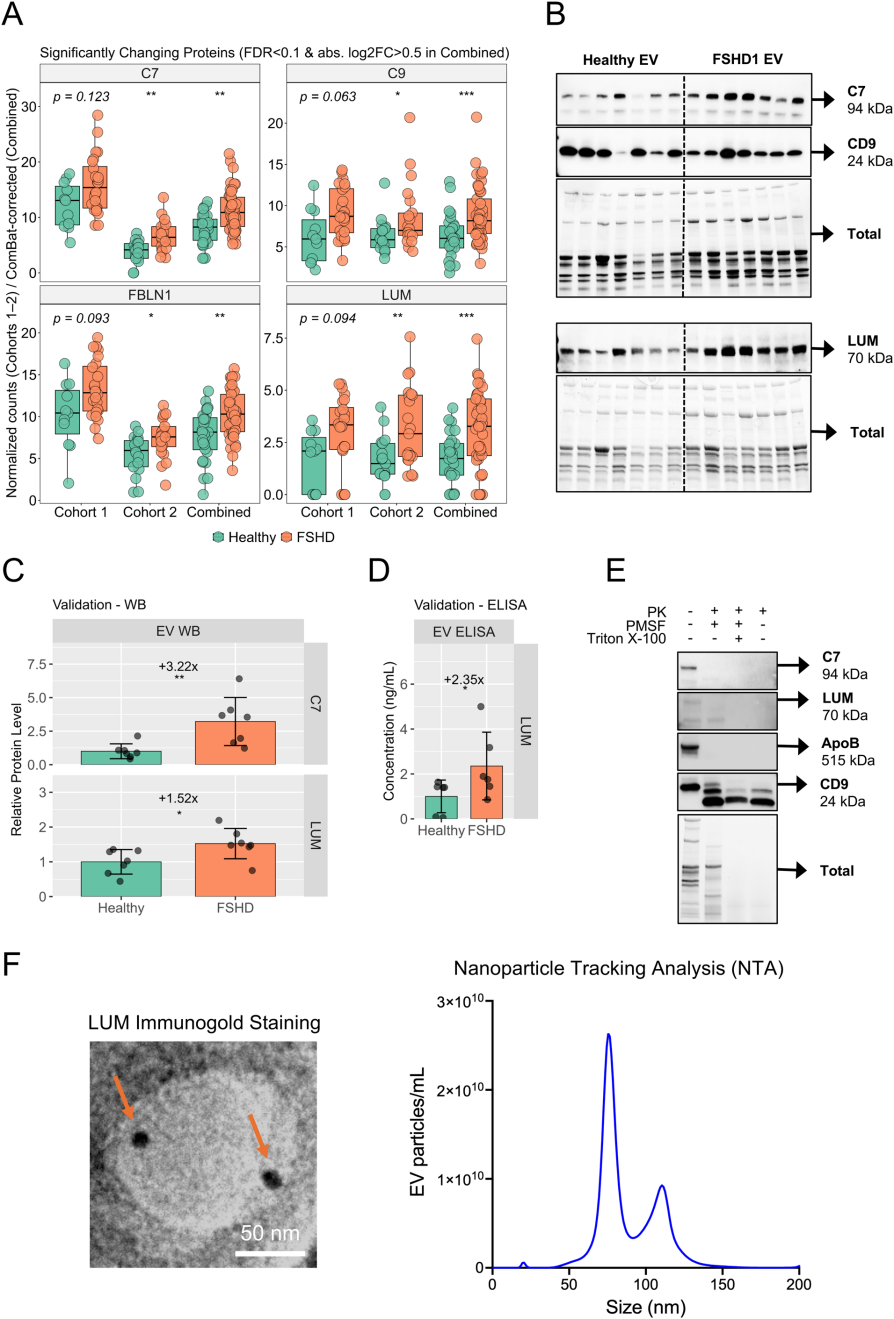
Validation of spectrometry data for C7 and LUM with secondary methods. (A) Spectral counts of top changing proteins (Combined Cohort, FDR < 0.1, |log2FC| > 0.5) in FSHD vs. Healthy across cohorts. *p < 0.05, **p < 0.01, ***p < 0.001 by Wald‐test. (B) Western blot verification of C7 and LUM levels. Tetraspanin CD9 was used as an EV marker. Stain‐free SDS‐PAGE gel was used to visualize total protein levels. (C) Quantification of Western blot data. C7 band intensity was normalized to total protein intensity. **p < 0.01 by Wilcoxon test. (D) ELISA verification of EV LUM levels. *p < 0.05 by Wilcoxon test. (E) Proteinase K (PK) treatment of plasma EV isolated from an FSHD1 patient (20 μg/mL PK for 1 h). Protease inhibitor PMSF (20 μM) is used to timely quench PK. 1% Triton X‐100 is used to lyse open EVs. ApoB is used as a corona layer control. (F) Transmission Electron Microscopy (TEM) of plasma EVs isolated from an FSHD1 patient. Immunogold staining detected positive LUM signals (gold arrows) on EV surface (Mean AuNP/EV: 1.40 ± 0.96; percent positive EVs: 54% ± 14%). The graph on the right shows EV size distribution and concentration measured by Nanoparticle Tracking Analysis (NTA). Uncropped Western blots can be found in Figure S8.

Next, we performed differential abundance analysis using the combined dataset, which comprised 45 FSHD1 patients and 28 healthy controls and identified four proteins significantly upregulated in FSHD EVs (|log2FC| > 0.5 and FDR < 0.1). These proteins were Lumican (LUM, log2FC = 0.928, *p* = 0.0006, FDR = 0.055), Complement component 7 (C7, log2FC = 0.706, *p* = 0.0012, FDR = 0.055), Complement component 9 (C9, log2FC = 0.532, *p* = 0.0003, FDR = 0.055), and Fibulin‐1 (FBLN1, log2FC = 0.531, *p* = 0.0011, FDR = 0.055) (Figures 2D, 3A and Table 2). KEGG analysis of the combined cohort was consistent with the single‐cohort analyses and highlighted the same enriched pathways (Figures S2F and 2E).

**TABLE 2:** Top 15 differentially abundant proteins (*p* < 0.05) in the Combined Cohort. Base Mean: Mean spectral counts in the healthy group. FC: Fold Change. Standard Error, *p* and FDR calculated with Wald Test. Age and sex were introduced as covariates.

Protein	Base Mean	FC in FSHD1	Std. Error	*p*	FDR
C9	7.858	1.447	0.150	0.0004	0.0538
LUM	2.534	1.903	0.270	0.0006	0.0538
CFB	14.951	1.309	0.118	0.0010	0.0538
FBLN1	9.331	1.445	0.163	0.0011	0.0538
C7	9.922	1.631	0.218	0.0012	0.0538
TNC	10.685	1.455	0.189	0.0042	0.1592
THBS4	9.157	1.390	0.169	0.0048	0.1592
C6	14.335	1.295	0.135	0.0057	0.1627
CLEC3B	1.619	2.112	0.412	0.0089	0.1926
CNDP1	2.559	0.496	0.390	0.0094	0.1926
HLA‐A	1.709	0.531	0.355	0.0101	0.1926
GPLD1	7.682	0.614	0.275	0.0104	0.1926
PLG	23.270	1.292	0.145	0.0109	0.1926
HPR	272.514	0.825	0.115	0.0154	0.2315
CLU	49.091	0.854	0.094	0.0156	0.2315

To better understand the biological significance of the differentially expressed proteins in segregating FSHD subjects, we calculated a composite *z*‐score for each subject in both cohorts using five FDR‐adjusted upregulated proteins in FSHD1 patients. We then ranked each participant based on the calculated composite *z*‐score (Figure 2F). As a result, most subjects (33/34) with a positive *z*‐score were FSHD1 patients. We did not observe cohort‐specific clustering, further suggesting that the two datasets were efficiently harmonized after batch correction (Figure 2F).

While all differential protein analyses comparing FSHD1 with healthy controls were adjusted for sex, we also performed sex‐stratified analyses in each cohort (Figure S4A–D) and the combined cohort (Figure S4E,F). In the combined analysis, no proteins met the stringent thresholds (|log2FC| > 0.5 and FDR < 0.1) among females (Figure S4E), whereas six did among males (Figure S4F): Desmoplakin (DSP), Complement component 9 (C9), Complement component 6 (C6), Tetranectin (CLEC3B), Fibulin‐1 (FBLN1), and Hornerin (HRNR). Moreover, two proteins that ranked among the top upregulated in the pooled analysis, LUM and C7, were also increased at nominal significance (*p* < 0.05) in both sex‐specific subsets of the combined cohort (Figure S4E,F). Together, these findings support consistent upregulation of LUM and C7 in FSHD1 and indicate that disease‐associated proteomic signatures are more pronounced in males.

### 3.3 The Differentially Enriched Protein Biomarkers Are Associated With EVs

Due to their consistent elevation in FSHD1 patients across cohorts and sexes, we focused on C7 and LUM (Figures 3A and S2E,F). EV identities were confirmed by using a panel of EV and contamination markers (Figure S5). We first validated the upregulations of C7 and LUM by Western blot in randomly selected FSHD1 patients and healthy controls (*N* = 7, Figure 3B). Tetraspanin CD9 was used as an EV marker. C7 and LUM levels were normalized to total protein levels (Figure 3C). Moreover, we also confirmed the enrichment of LUM in FSHD EVs using a commercial ELISA kit for LUM (Figure 3D).

As plasma EVs are heavily decorated with a protein corona and may carry co‐isolate non‐vesicular proteins, we treated the EVs with Proteinase K (PK), which digests surface‐exposed proteins without crossing the lipid bilayer. PK treatment of EVs diminished the C7 and LUM signals by Western blot, while the transmembrane protein CD9 was only truncated (Figure 3E), suggesting that C7 and LUM are predominantly surface‐associated or components of the EV protein corona rather than luminal EV cargo. Next, we investigated this possibility. While complement proteins are known constituents of the EV corona [44], LUM's localization in relation to EVs is still elusive. Transmission electron microscopy (TEM) with gold‐labeled antibodies showed LUM localization on the EV surface, with vesicles displaying at least one LUM‐positive signal per EV particle (Mean AuNP/EV: 1.40 ± 0.96; percent positive EVs: 54% ± 14%) (Figure 3F).

### 3.4 Surface‐Bound EV Lumican Is Glycosylated

Lumican (LUM) is a highly glycosylated protein whose molecular weight increases from its 38‐kDa core depending on tissue context [34, 45]. In plasma EVs, we consistently detected a 70‐kDa band, indicating glycosylated LUM. PNGase F treatment removed N‐linked glycans from EV‐associated LUM, eliminating the heavily glycosylated ~70‐kDa band and yielding a band at the expected ~38‐kDa core size together with a prominent ~30‐kDa fragment, suggestive of a shorter or truncated LUM isoform (Figure S6). Notably, in striated muscle, glycosylated LUM has been linked to disease‐related tissue remodeling, including protection against skeletal muscle loss and myogenesis stimulation in sarcopenic muscles, as well as accumulation with fibrillar collagen in fibrotic hypertrophic myocardia [46, 47].

### 3.5 EV Proteomic Signatures Correlate With FSHD1 Clinical Outcome Measures

**FIGURE 4:**
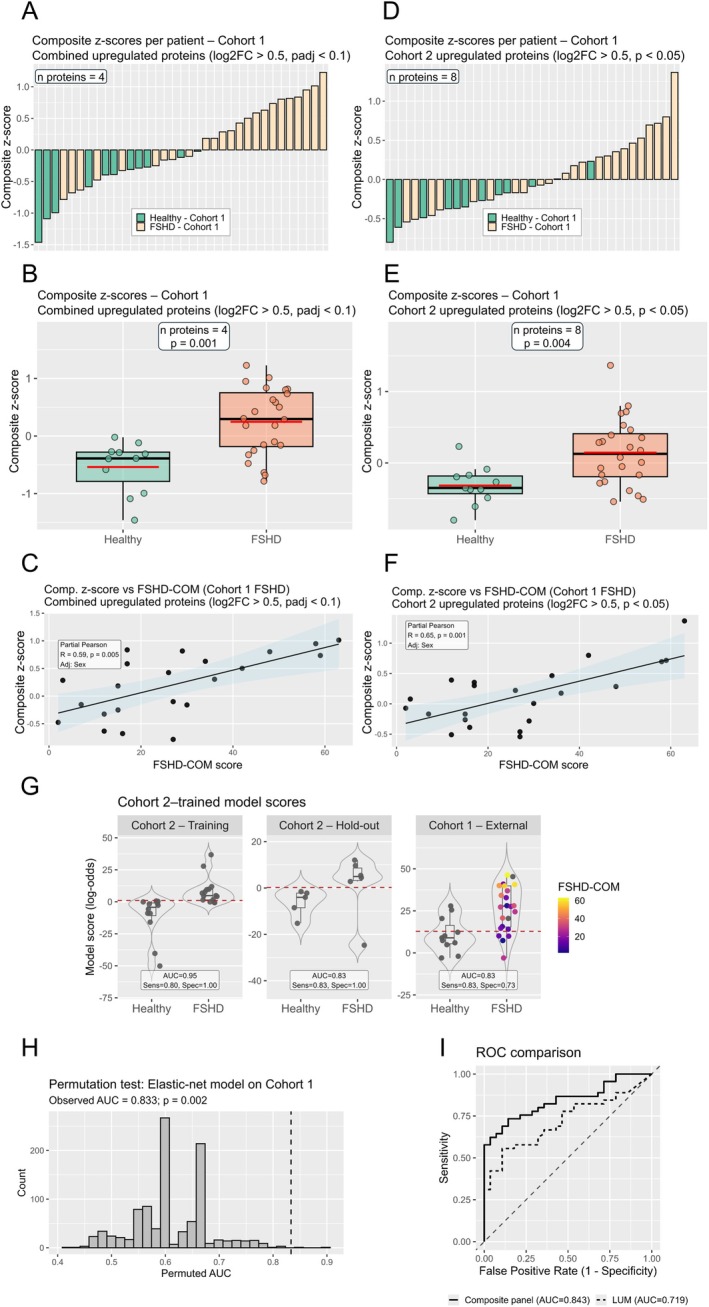
Upregulated proteins in FSHD1 patients significantly correlate with clinical outcome measures of patients in Cohort 1. (A) Composite *z*‐scores of a panel of four upregulated proteins (log2FC > 0.5, FDR < 0.1 in Combined Cohort) were calculated and evaluated in FSHD1 patients and healthy controls of Cohort 1. All participants of Cohort 1 were ranked based on composite *z*‐scores for these select proteins. (B) Boxplots of composite *z*‐scores from (A). Points represent individual samples; boxes show interquartile range with median; red lines indicate means. *p* calculated using the Wilcoxon rank‐sum test. (C) Pearson R correlation of disease severity (as evaluated by FSHD‐COM score) with composite *z*‐score (FDR < 0.1 in Combined Cohort) in Cohort 1. (D) Composite *z*‐scores of a panel of eight upregulated proteins (log2FC > 0.5, *p* < 0.05 in Cohort 2) are calculated and evaluated in FSHD1 patients and healthy controls of Cohort 1. All participants of Cohort 1 are ranked based on composite *z*‐scores for these select proteins. (E) Boxplots of composite *z*‐scores from (D). Points represent individual samples; boxes show interquartile range with median; red lines indicate means. *p* calculated using the Wilcoxon rank‐sum test. (F) Pearson R correlation of disease severity (as evaluated by FSHD‐COM score) with composite *z*‐score (*p* < 0.05 in Cohort 2) Cohort 1. All Pearson R correlations with FSHD‐COM scores are adjusted for sex (Partial Pearson). Confidence intervals (95%) for each correlation are indicated by blue shade. (G) An elastic‐net–regularized logistic regression model trained on 75% of Cohort 2 identified LUM and C7 upregulation and CNDP1 downregulation as key predictors of FSHD1. The resulting model showed strong discrimination in the Cohort 2 hold‐out set and generalized well to the independent Cohort 1 validation set. Violin plots show model scores (linear predictor) for each dataset with Cohort 1 points colored by FSHD‐COM severity. Higher model scores correspond to higher predicted probability of FSHD1. AUC: Area Under Curve. Sensitivity (sens.) and specificity (spec.) scores indicate model's ability to predict healthy controls and FSHD1 patients, respectively. Scale: 0–1 where 1.0 is perfect. (H) Permutation testing of the elastic‐net model in Cohort 1. Histogram shows AUC values from 1000 label permutations; dashed line indicates the observed AUC. The permutation *p*‐value reflects the proportion of permuted models with AUC ≥ observed. (I) ROC curves for Lumican (LUM) alone and the composite biomarker panel (LUM, C7, C9, FBLN1). The composite panel shows higher AUC, indicating improved discrimination. The dashed diagonal represents random classification.

We next examined whether the EV biomarkers correlated with clinical outcome measures. Correlations with outcome measures were adjusted to age and/or sex according to their impact on the outcome measure (Table S6). First, we used a panel of five proteins upregulated in the combined cohort (log2FC > 0.5, FDR < 0.1). We calculated a composite *z*‐score across this panel and evaluated their relative levels in Cohort 1, where clinical data is available. This composite metric separated FSHD1 patients from healthy controls (mean *z* = 0.247, and −0.316, respectively, *p* < 0.001, Figure 4A,B) and correlated significantly with disease severity, as reflected by the FSHD‐COM score (sex‐adjusted partial Pearson *R* = 0.59, *p* = 0.005; Figure 4C). We also repeated this analysis using only a panel of upregulated (log2FC > 0.5, *p* < 0.05) proteins obtained from the second cohort. Composite *z*‐scores of this new panel also segregated FSHD1 patients from healthy controls (mean *z* = 0.316, and −0.145 respectively, *p* < 0.004, Figure 4D,E) and correlated with disease severity (sex‐adjusted partial Pearson *R* = 0.65, *p* = 0.001; Figure 4F). Finally, we set out to conduct a similar analysis using the signature obtained from Cohort 1 and evaluated in Cohort 2. As Cohort 1 only contained a single protein upregulated in FSHD1 patients (log2FC > 0.5, *p* < 0.05), we proceeded with evaluating the composite *z*‐scores of downregulated proteins. Compared to healthy controls, FSHD1 patients in Cohort 2 had lower composite *z*‐scores, but the difference did not reach statistical significance (mean *z* = 0.183, and −0.148, respectively, *p* = 0.243, Figure S7A,B). To further assess whether these proteomic patterns could be used for disease classification, we applied a machine‐learning approach. An elastic‐net regularized logistic regression model was trained on 75% of Cohort 2 samples using all detectable proteins adjusted for age and sex. The model identified LUM and C7 upregulation, and CNDP1 downregulation, as the strongest predictors of FSHD1 status. Generalized Linear Model (GLM) coefficients for LUM, C7, and CNDP1, which indicate how much each protein contributes to the FSHD1 likeness score were + 0.568, +0.441, and −0.0515, respectively. The model showed strong performance in both the internal hold‐out set (AUC = 0.87, sensitivity = 0.83, specificity = 1.00) and the independent external validation cohort (Cohort 1; AUC = 0.79, sensitivity = 0.71, specificity = 0.91; Figure 4G). For reference, an AUC of 1.0 indicates perfect discrimination between FSHD1 and healthy samples, while sensitivity reflects the proportion of true FSHD1 cases correctly identified and specificity reflects the proportion of healthy controls correctly classified. Importantly, in the external cohort, individuals with higher FSHD‐COM scores exhibited higher machine learning model scores, demonstrating that the quantitative output of the prediction reflects clinical status. Permutation testing of the elastic‐net classifier by repeated label shuffling confirmed that the observed performance on the external Cohort 1 validation set exceeded random expectation (AUC = 0.833, permutation *p* = 0.002, Figure 4H). Complementary ROC analysis demonstrated moderate discriminatory ability for Lumican alone (AUC = 0.719), whereas the composite biomarker panel showed stronger discrimination (AUC = 0.843, Figure 4I). Altogether, these results highlight an association between circulating EV protein signatures and clinical severity in FSHD1.

**FIGURE 5:**
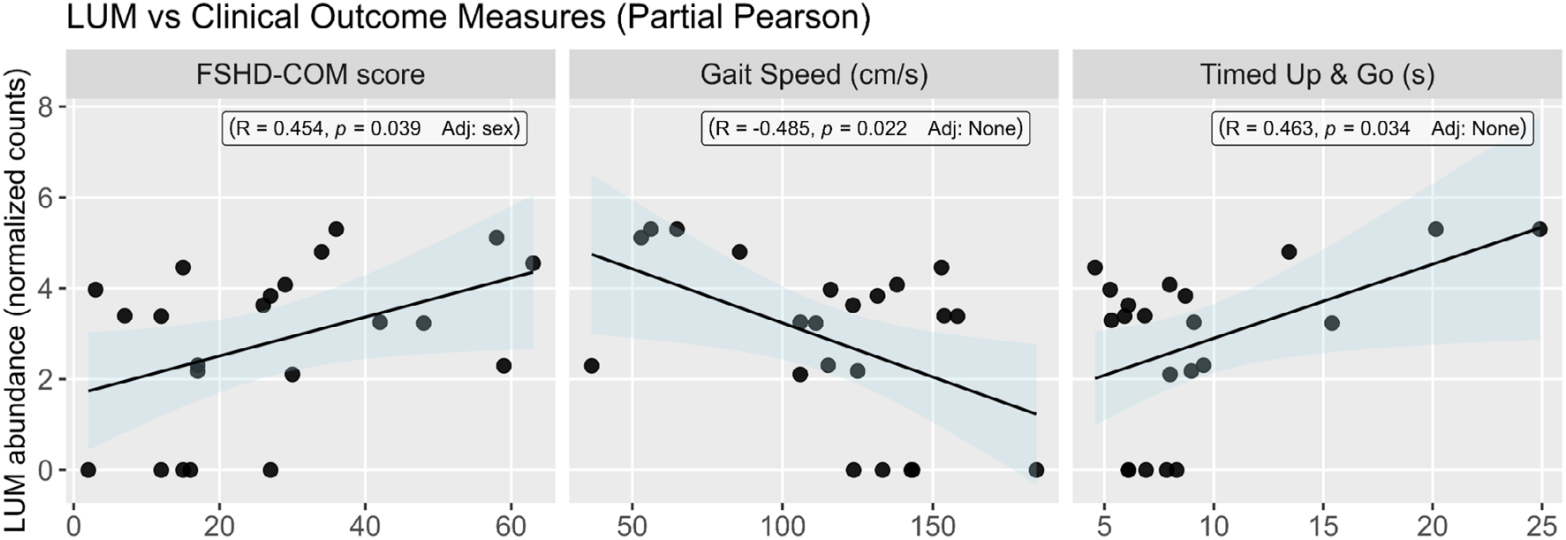
Correlation of LUM with clinical outcome measures. Pearson R correlations of FSHD‐COM score, Self‐selected Gait Speed and Timed Up and Go with LUM levels in Cohort 1. Pearson R correlations with FSHD‐COM score are adjusted for sex. Confidence intervals (95%) for each correlation are indicated by blue shade.

Finally, we performed correlation analysis of individual proteins with other continuous clinical outcome measures. Among these, LUM was significantly correlated with multiple outcome measures, including FSHD‐COM (sex‐adjusted Partial Pearson *R*: 0.454, *p*: 0.039), self‐selected gait speed time (Pearson *R*: −0.485, *p*: 0.022), and timed up and go (Pearson R: 0.463, *p*: 0.034) (Figure 5 and Table S6).

## 4 Discussion

In FSHD clinical trials, outcome measures, including patient‐reported clinical outcomes, imaging, biopsy‐based, and functional assessments, often lack the sensitivity needed to detect subtle changes in affected tissues and can be invasive, costly, time‐consuming, and variable across clinical sites. This creates major challenges for patient stratification and can delay therapeutic development. Circulating molecular biomarkers offer an attractive complementary approach as non‐invasive, objective endpoints that could facilitate earlier detection of disease manifestation, predict disease progression, and assess therapeutic efficacy and durability. Hence, we explored the proteome of plasma‐derived EVs in FSHD1 patients and healthy controls, with the rationale that EV‐associated cargo and surface‐corona composition may better reflect ongoing pathology than crude plasma alone, which is expected to increase the sensitivity of detecting subtle changes.

Here, we present the first unbiased, cross‐cohort proteomic profiling of plasma EVs in FSHD1, analyzing 45 patients and 28 healthy controls distributed across two independent cohorts. A clear separation of FSHD patients was revealed using supervised multivariate modeling, and differential abundance tests provided an FSHD EV signature dominated by ECM and complement‐related proteins [42]. After harmonizing cohorts with ComBat while preserving disease effects, four proteins met stringent criteria for upregulation in the combined cohort (LUM, C7, C9, and FBLN1), and pathway enrichment consistently highlighted complement activation and ECM remodeling. FSHD muscle pathology is characterized by chronic inflammation and ECM remodeling, processes that are closely linked to complement activation. Complement signaling can influence immune cell recruitment and tissue repair, while ECM components regulate fibrotic responses in injured muscle [48, 49, 50]. Hence, our findings support the concept that EV proteomics can capture biologically meaningful and clinically relevant features of FSHD1. An important modifier of these EV‐associated proteomic signatures was sex. In sex‐stratified analyses, disease‐associated proteomic differences were more pronounced in males, whereas no proteins met FDR threshold among females (both have a median age of 48) (Figure S4E,F). Despite this difference in statistical strength, key biomarkers identified in the pooled analysis, LUM and C7, showed consistent directionality and remained nominally increased in both sexes. This pattern suggests that while the magnitude of proteomic alterations may differ by sex, core components of the disease‐related signature are preserved. Sex‐related differences are a recognized feature of FSHD, with males on average exhibiting greater disease severity, which may in turn accentuate systemic inflammatory and extracellular matrix remodeling processes captured by circulating EVs [51, 52]. Nevertheless, these findings should be interpreted with caution, as sex‐stratified analyses reduce statistical power and our female subgroup may be underpowered to detect subtler effects. Future studies with larger cohorts will be required to rigorously define sex‐specific EV proteomic signatures and to determine whether distinct biomarker thresholds or panels may be needed for optimal clinical application across sexes.

A key observation from our study is that plasma EV‐associated proteins in FSHD predominantly reflect inflammatory/ECM remodeling processes rather than bona fide skeletal muscle proteins. Unlike dystrophinopathies where muscle destruction is extensive, FSHD muscle damage can be patchy and slower in progression, which may reduce the sensitivity of detecting clear muscle‐specific EV signatures in plasma. Nevertheless, EV “leakage” into the circulation may increase upon injury, and muscular dystrophy is expected to enhance the contribution of muscle‐derived EVs. These vesicles carry proteins within their lumen and on their surface, where surface‐associated proteins form a stable and functionally relevant protein corona that influences EV density, integrity, and biological interactions [53, 54]. Our proteinase K experiments for C7 and LUM and immunogold TEM for LUM support the idea that these two biomarkers are largely surface‐exposed and/or corona‐associated in plasma EV preparations. This suggests that EV‐associated biomarkers in circulation may not be exclusively confined to the EV lumen.

The complement signature in FSHD1 patients we observed is particularly relevant. Mild elevations of complement components (e.g., C3 and C4b) in crude plasma have been reported in FSHD and complement activation has been suggested as an early feature of pathological activity in STIR‐positive muscle [21, 55]. Our results extend these observations by showing that a distinct set of complement proteins (including C7 and C9, and in male‐stratified analyses C6, which are components of the complement membrane attack complex, MAC) is differentially enriched in plasma EVs from FSHD1 patients. While complement proteins are well‐established components of the plasma EV protein corona [44], their enrichment on EV surfaces may help explain why prior plasma studies often report subtle effects: EV surfaces can concentrate certain complement factors, potentially enhancing sensitivity to FSHD‐specific complement activation compared to crude plasma measurements. Taken together, our data suggest a model in which systemic immune activation and tissue ECM remodeling in FSHD are reflected in EVs. Similar EV‐associated remodeling‐driven changes in EV surface and protein‐corona composition have been described across fibrotic, cardiovascular, and inflammatory diseases [42]. These changes are shaped by the extracellular milieu rather than selective loading of tissue‐specific intracellular cargo [44, 53, 54, 56].

Within this remodeling‐centric EV framework, LUM emerged as a particularly consistent and clinically relevant disease‐associated protein across our datasets. It was significantly upregulated in Cohort 2 and in the combined cohort, and showed apparent clinical associations in Cohort 1, the cohort from which clinical outcome measures were obtained. EV‐associated LUM correlated significantly with FSHD‐COM, self‐selected gait speed time, and timed up and go, indicating that this prospective biomarker is not only elevated in FSHD1 patients, but also correlates with clinical severity and some continuous clinical outcome measures. Biologically, this aligns with the role of LUM as an ECM proteoglycan enriched in fibrotic tissues and implicated in inflammatory processes [45, 46, 47]. Notably, prior work investigating the power of imaging‐based biomarkers to predict FSHD signatures also investigated the role of infiltrating non‐muscle cells in DUX4 signature expression [57]. Accordingly, RNA deconvolution analysis identified a LUM‐positive fibro‐adipogenic progenitor (FAP) subtype whose abundance and transcriptome signature correlated with the activation of the DUX4 transcriptional program [57]. FAPs are muscle‐resident mesenchymal cells known to accumulate in DUX4‐expressing muscles and thought to contribute to muscle fibrosis and fat replacement in FSHD^57^. Furthermore, proteomic analyses of *biceps brachii* muscle in FSHD1 patients revealed that while LUM was decreased in mild FSHD1 patients, both LUM and FBLN1 were significantly upregulated in severe FSHD1 patients, correlating with the accumulation of collagen and molecules promoting ECM interaction and fibrillogenesis [58]. Our findings extend these tissue‐level signatures and position EV‐associated LUM as a surrogate circulating biomarker of muscle ECM remodeling rather than direct muscle injury. While the mechanistic link remains to be established, prior associations between ECM disruption, complement activation, and MAC deposition in FSHD muscle support the biological relevance of this EV‐based signal, which may capture a remodeling‐associated disease state that precedes or evolves independently of overt myofiber injury [59, 60]. Our detection of MAC components on circulating EVs is consistent with this proposed model.

Our results also suggest that combinatorial EV signatures may distinguish disease state better than single biomarkers. Composite *z*‐scores computed from panels of upregulated proteins separated most FSHD1 patients from controls and correlated with disease severity in Cohort 1. In addition, an elastic‐net classifier trained in Cohort 2 was validated in Cohort 1, where LUM and C7 upregulation and CNDP1 downregulation emerged as dominant predictors.

This study has limitations that need to be addressed in future studies. First, our cohorts are cross‐sectional, preventing interpretation about patient‐specific biomarker dynamics over time. Second, while our data suggest that this EV‐associated corona biology is informative, saturation of proteomic data with corona proteins may mask the detection of luminal EV proteins by mass spectrometry, a method which is usually biased toward high abundance molecules. Third, our study partially relies on ComBat batch correction of separately analyzed proteomic datasets, as previously done [61]. However, we applied this batch correction at the protein level rather than at the peptide or precursor level because it is the most robust approach for label‐free proteomic data [62]. Accordingly, ComBat correction effectively mitigated batch‐specific technical effects (PCA; Figure 2A) while preserving disease‐associated biological signatures (PLS‐DA; Figure 2B). Nevertheless, it cannot fully account for complex or non‐linear biases inherent to proteomic measurements, and optimal batch‐correction strategies for proteomics remain only partly explored [63]. Considering these limitations, it is reassuring that LUM associations remain consistent across single cohorts: LUM drives PLS‐DA separation in both cohorts, correlates with available clinical measures in Cohort 1, and is significantly upregulated in Cohort 2.

Our next steps would be to validate our findings in a third, larger FSHD cohort using a targeted cross‐sectional design, and then extend to a longitudinal study to determine whether our EV protein biomarkers accurately track disease progression, using high‐sensitivity detection platforms. Finally, it would be of high interest to determine the response of our EV protein biomarkers to therapy by evaluating their modulation in longitudinal plasma samples collected before and after therapeutic intervention. This will be an essential benchmark for clinical trial readiness and the validation of our EV proteins as minimally invasive surrogate molecular biomarkers for FSHD.

Overall, our study provides the first cross‐cohort, unbiased evidence that plasma EV proteomes in FSHD1 patients carry a reproducible signature of complement activation and ECM remodeling, with LUM as a particularly consistent and clinically associated marker. These findings provide a rationale for targeted validation of circulating EV‐based proteins in larger FSHD1 cohorts to assess their potential clinical relevance as minimally invasive surrogate biomarkers for patient stratification and disease monitoring.

## Author Contributions

M.B.B., and N.Y.S. contributed to the conception and design of the study; M.B.B., J.S.N., C.K.N., K.T.N., X.Y.R., J.D., E.R., J.M.S., R.T., S.Q.H., and N.Y.S. contributed to the acquisition and analysis of data; J.M.S., R.T., and K.M.F. contributed to the clinical aspect of the study and to the collection of clinical samples; M.B.B., and N.Y.S. contributed to the drafting, reviewing and editing of the manuscript, and to the preparation of the figures.

## Funding

This work was supported by Nationwide Children’s Hospital, The Chris Carrino Foundation for FSHD to N.Y.S. and M.B.B., Friends of FSH Research to M.B.B., the Eunice Kennedy Shriver National Institute of Child Health & Human Development of the National Institutes of Health: P50HD117373 and the National Center for Advancing Translational Sciences of the National Institutes of Health: R21TR005565. The content is solely the responsibility of the authors and does not necessarily represent the official views of the National Institutes of Health.

## Conflicts of Interest

The authors declare no conflicts of interest.

## Supporting information


**Figure S1:** Permutation testing of PLS‐DA models in individual cohorts. (A,B) Histograms showing the null distributions of cross‐validated PLS‐DA error rates generated from 1000 random label permutations for Cohort 1 (A), Cohort 2 (B). The dashed vertical line marks the observed error rate of the corresponding model. Permutation *p*‐values indicate the proportion of permuted models with error rates equal to or lower than the observed model.

**Figure S2:** Differential protein levels in plasma EVs of FSHD1 patients and healthy controls adjusted for age and sex. (A,B) Volcano plots of the two independent cohorts. FDR < 0.1, *p* < 0.05 and absolute log2FC > 0.5 (dashed lines) were used as a cutoff for significance. Labeled are proteins that met the cutoff criteria (see also Tables S4and S5). (C,D) Corresponding heatmaps of relative levels (i.e., *z*‐scores) displaying all proteins that are significantly changing using nominal *p* < 0.05. For Cohort 1, patients were listed in order of disease severity based on FSHD‐COM score. Bold: FDR < 0.1. (E) Lollipop plot illustrating significantly changing proteins in each cohort. Absolute fold changes are labeled on the plot. *p* is calculated with Wald‐test. (F) Enriched KEGG pathways (Bonferroni corrected *p* < 0.05) conducted with DAVID software. *p* is calculated with Wald‐test.

**Figure S3:** Permutation testing of PLS‐DA models in the combined batch‐corrected cohort. Histograms showing the null distributions of cross‐validated PLS‐DA error rates generated from 1000 random label permutations. The dashed vertical line marks the observed error rate of the corresponding model. Permutation *p*‐values indicate the proportion of permuted models with error rates equal to or lower than the observed model.

**Figure S4:** Differential protein levels in plasma EVs of FSHD1 patients and healthy controls separated by sex, adjusted for age. Volcano plots of Cohort 1 (A,B), Cohort 2 (C,D), and Combined Cohort (E,F). Desmoplakin (DSP): log2FC = 4.534, *p* = 0.0002, FDR = 0.057), C9 (log2FC = 0.893, *p* = 0.0006, FDR = 0.078); Complement component 9 (C9): log2FC = 0.893, *p* = 0.0006, FDR = 0.078; Tetranectin (CLEC3B): log2FC = 2.545, *p* = 0.0015, FDR = 0.078; Fibulin‐1 (FBLN1): log2FC = 0.811, *p* = 0.0016, FDR = 0.078; Hornerin (HRNR): log2FC = 2.715, *p* = 0.002, FDR = 0.078; Lumican (LUM): female log2FC = 0.678, *p* = 0.040; male log2FC = 1.398, *p* = 0.009; C7: female log2FC = 0.621, *p* = 0.020; male log2FC = 0.877, *p* = 0.020. In volcano plots, FDR < 0.1, *p* < 0.05 and absolute log2FC > 0.5 (dashed lines) were used as a cutoff for significance.

**Figure S5:** Plasma EV identity confirmed by Western Blot. (A) Uncropped Western blot for Calnexin. (B) Uncropped Western blot for Flotillin. (C) Uncropped Western blot for TSG101. (D) Uncropped Western blot for CD9. CL: HEK293 cell lysis.

**Figure S6:** Treatment of plasma EVs with PNGase F reduced the observed molecular weight of LUM. 25 μg of EV proteins were incubated with (i) PBS at 4°C for one‐hour (EV control), (ii) PBS at 50°C for one‐hour (heat control) (iii) PNGase F mix at 50°C for one‐hour. Stain‐free SDS‐PAGE gel was used to visualize total protein levels.

**Figure S7:** Cross‐cohort evaluation of Cohort 1 downregulated protein signature in Cohort 2. (A) Composite *z*‐scores of a panel of five downregulated proteins (log2FC > −0.5, *p* < 0.05 in Cohort 1) are calculated and evaluated in FSHD1 patients and healthy controls of Cohort 2. All participants of Cohort 2 are ranked based on composite *z*‐scores for these select proteins. (B) Boxplots of composite *z*‐scores from (A). Points represent individual samples; boxes show interquartile range with median; red lines indicate means. *p* calculated using the Wilcoxon rank‐sum test.

**Figure S8:** Uncropped Western blot images. (A) Uncropped images of Western blots shown in Figure 3B. Cropped areas are indicated. (B) Uncropped images of Western blots shown in Figure 3E. Samples run in duplicates. Cropped areas are indicated.

**Table S1:** Mass spectrometry counts and clinical data.

**Table S2:** Variable Importance in Projection (VIP) scores for each cohort.

**Table S3:** Differential protein abundance analysis between FSHD1 patients and healthy controls in each cohort.

**Table S4:** Differentially abundant proteins (*p* < 0.05) in Cohort 1. Base Mean: Mean spectral counts in the healthy group. FC: Fold Change. Standard Error, *p* and FDR calculated with Wald Test. Age and sex were introduced as covariates.

**Table S5:** Differentially abundant proteins (*p* < 0.05) in Cohort 2. Base Mean: Mean spectral counts in the healthy group. FC: Fold Change. Standard Error, *p* and FDR calculated with Wald Test. Age and sex were introduced as covariates.

**Table S6:** Correlations of five proteins upregulated in the combined cohort with outcome measures.

## Data Availability

All datasets generated during this study are available in the article and in the Supporting Information files. All datasets, codes and materials are available from the corresponding author on reasonable request.
