# Supplementary figures and images for: Plasma EV Proteomics Identifies ECM Remodeling and Inflammatory Proteins LUM and C7 as Candidate Biomarkers in FSHD
Source: Ann Clin Transl Neurol. 2026 May 20:10.1002/acn3.70435. Online ahead of print. doi: 10.1002/acn3.70435 (PMC13394447; doi:10.1002/acn3.70435)

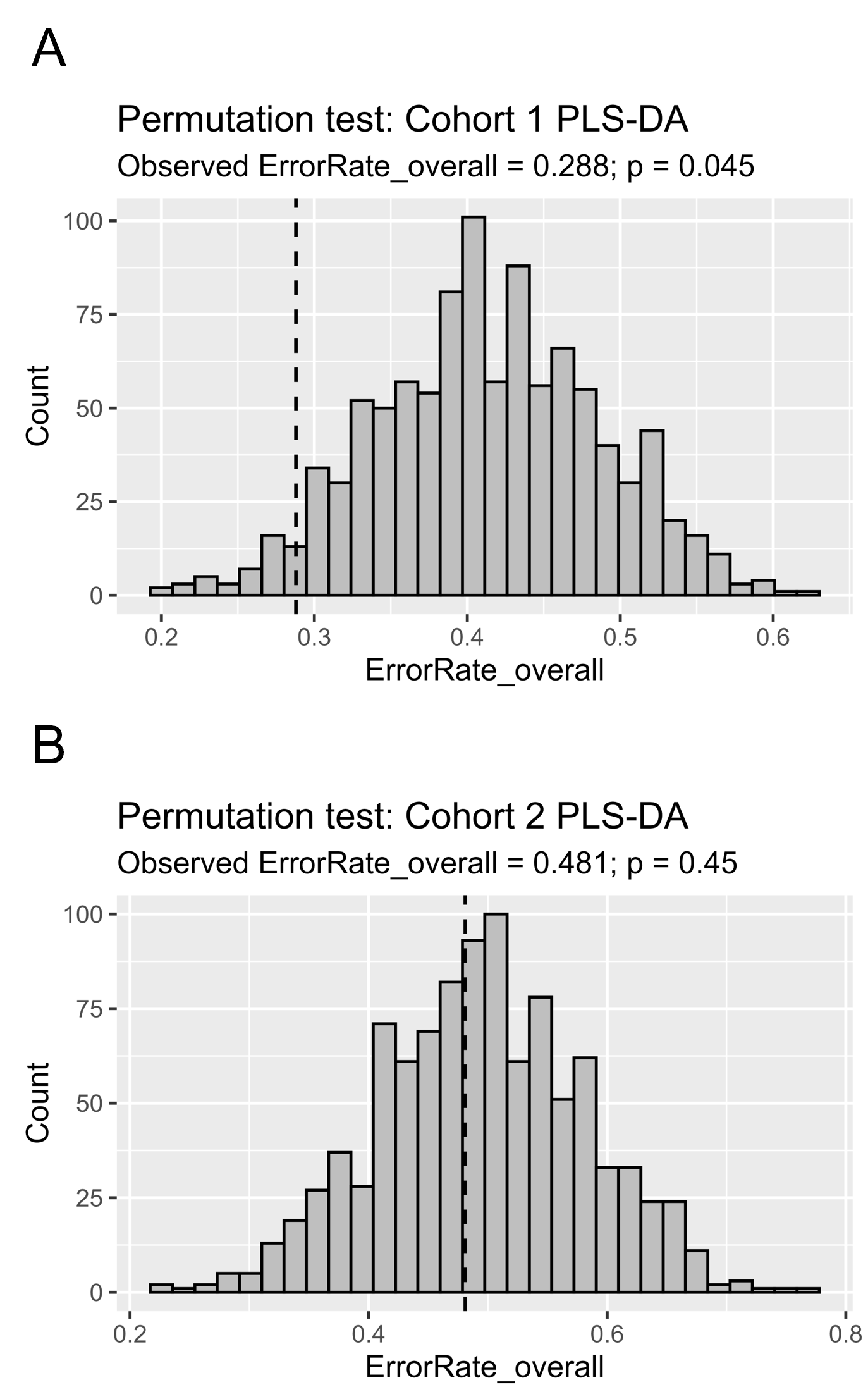

Supplement: Supplementary file 1 — Figure S1: Permutation testing of PLS‐DA models in individual cohorts. (A,B) Histograms showing the null distributions of cross‐validated PLS‐DA error rates generated from 1000 random label permutations for Cohort 1 (A), Cohort 2 (B). The dashed vertical line marks the observed error rate of the corresponding model. Permutation p‐values indicate the proportion of permuted models with error rates equal to or lower than the observed model. [file ACN3-9999-0-s004.tif]

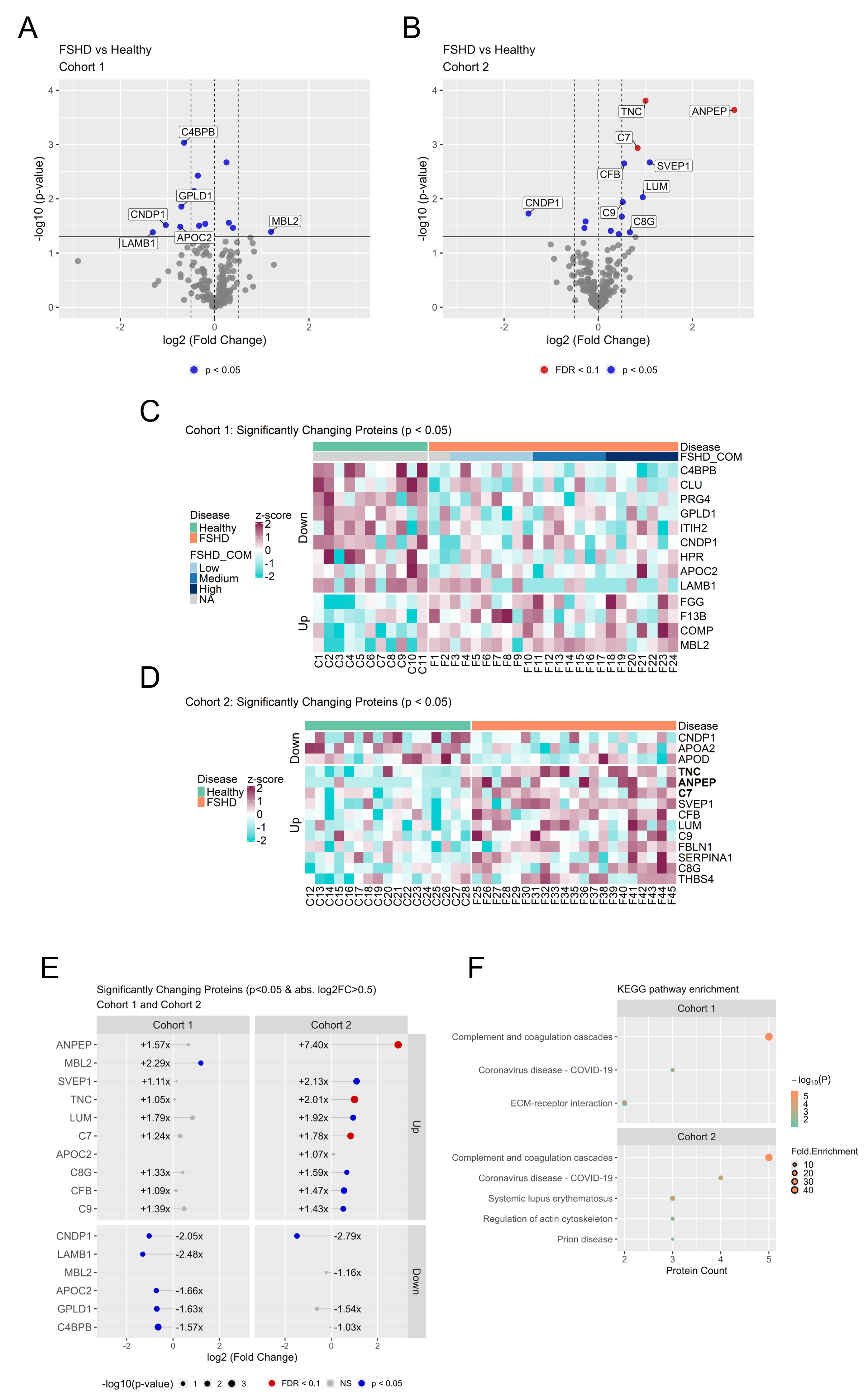

Supplement: Supplementary file 2 — Figure S2: Differential protein levels in plasma EVs of FSHD1 patients and healthy controls adjusted for age and sex. (A,B) Volcano plots of the two independent cohorts. FDR < 0.1, p < 0.05 and absolute log2FC > 0.5 (dashed lines) were used as a cutoff for significance. Labeled are proteins that met the cutoff criteria (see also Tables S4and S5). (C,D) Corresponding heatmaps of relative levels (i.e., z‐scores) displaying all proteins that are significantly changing using nominal p < 0.05. For Cohort 1, patients were listed in order of disease severity based on FSHD‐COM score. Bold: FDR < 0.1. (E) Lollipop plot illustrating significantly changing proteins in each cohort. Absolute fold changes are labeled on the plot. p is calculated with Wald‐test. (F) Enriched KEGG pathways (Bonferroni corrected p < 0.05) conducted with DAVID software. p is calculated with Wald‐test. [file ACN3-9999-0-s005.tif]

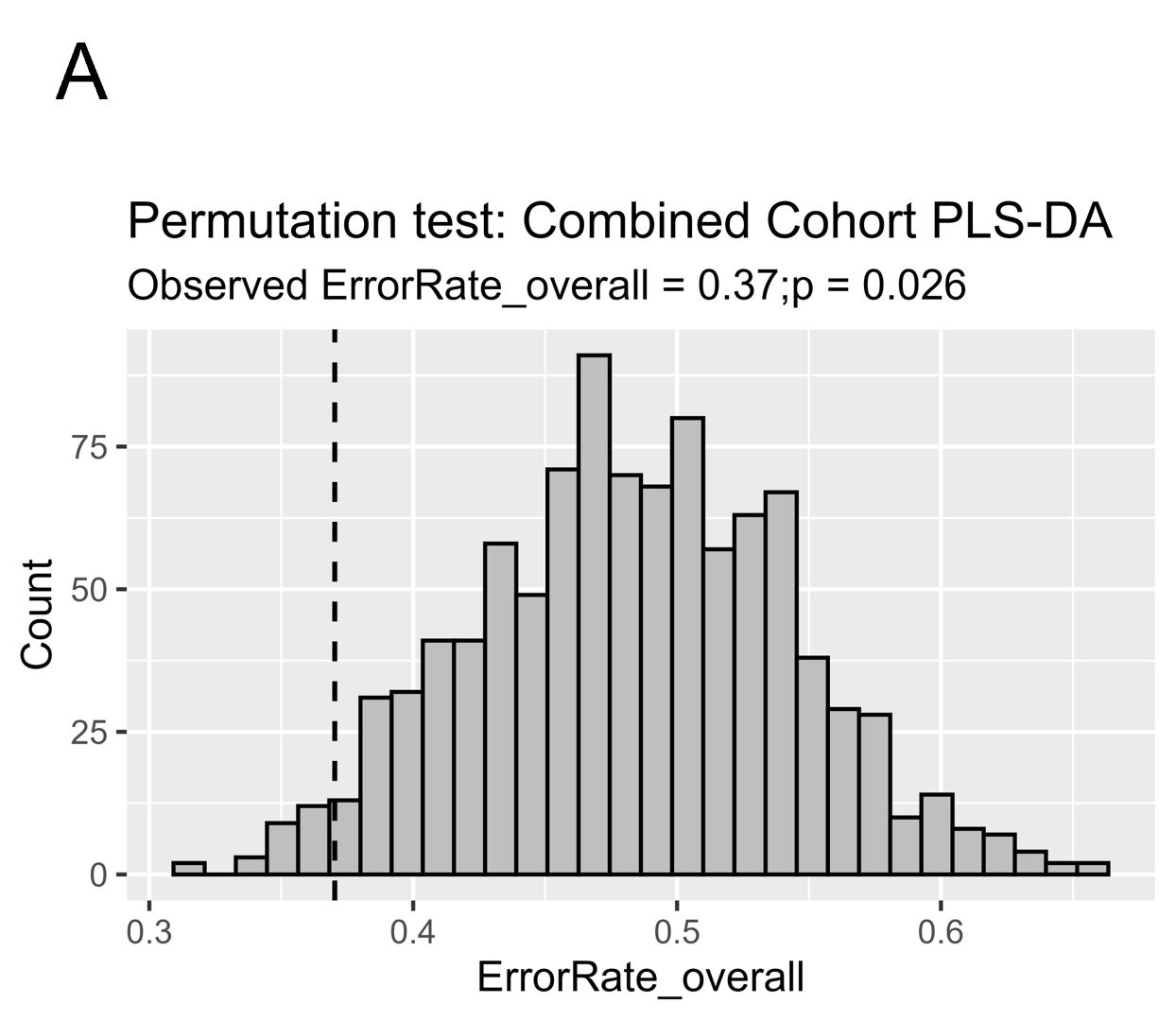

Supplement: Supplementary file 3 — Figure S3: Permutation testing of PLS‐DA models in the combined batch‐corrected cohort. Histograms showing the null distributions of cross‐validated PLS‐DA error rates generated from 1000 random label permutations. The dashed vertical line marks the observed error rate of the corresponding model. Permutation p‐values indicate the proportion of permuted models with error rates equal to or lower than the observed model. [file ACN3-9999-0-s010.tif]

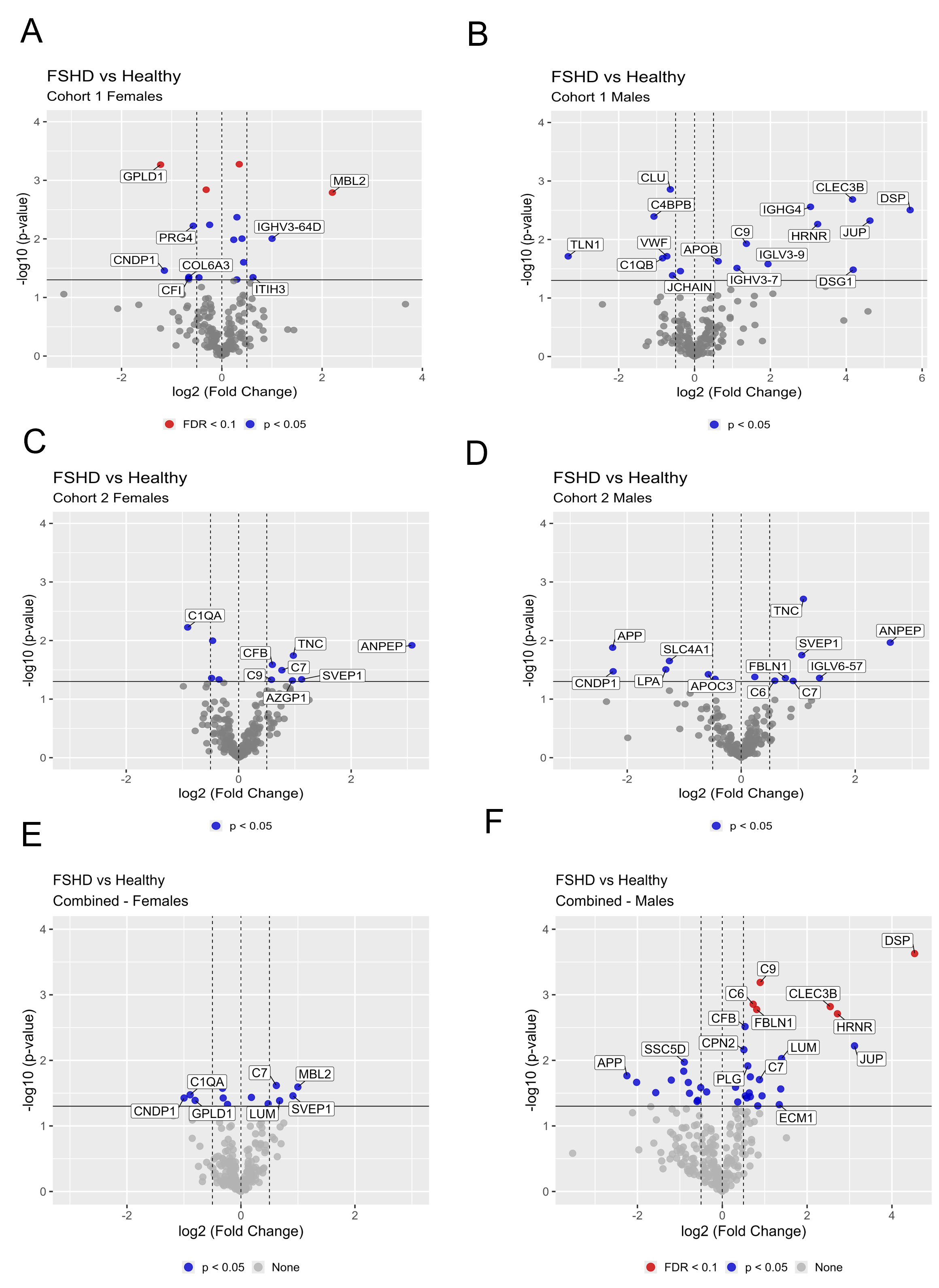

Supplement: Supplementary file 4 — Figure S4: Differential protein levels in plasma EVs of FSHD1 patients and healthy controls separated by sex, adjusted for age. Volcano plots of Cohort 1 (A,B), Cohort 2 (C,D), and Combined Cohort (E,F). Desmoplakin (DSP): log2FC = 4.534, p = 0.0002, FDR = 0.057), C9 (log2FC = 0.893, p = 0.0006, FDR = 0.078); Complement component 9 (C9): log2FC = 0.893, p = 0.0006, FDR = 0.078; Tetranectin (CLEC3B): log2FC = 2.545, p = 0.0015, FDR = 0.078; Fibulin‐1 (FBLN1): log2FC = 0.811, p = 0.0016, FDR = 0.078; Hornerin (HRNR): log2FC = 2.715, p = 0.002, FDR = 0.078; Lumican (LUM): female log2FC = 0.678, p = 0.040; male log2FC = 1.398, p = 0.009; C7: female log2FC = 0.621, p = 0.020; male log2FC = 0.877, p = 0.020. In volcano plots, FDR < 0.1, p < 0.05 and absolute log2FC > 0.5 (dashed lines) were used as a cutoff for significance. [file ACN3-9999-0-s013.tif]

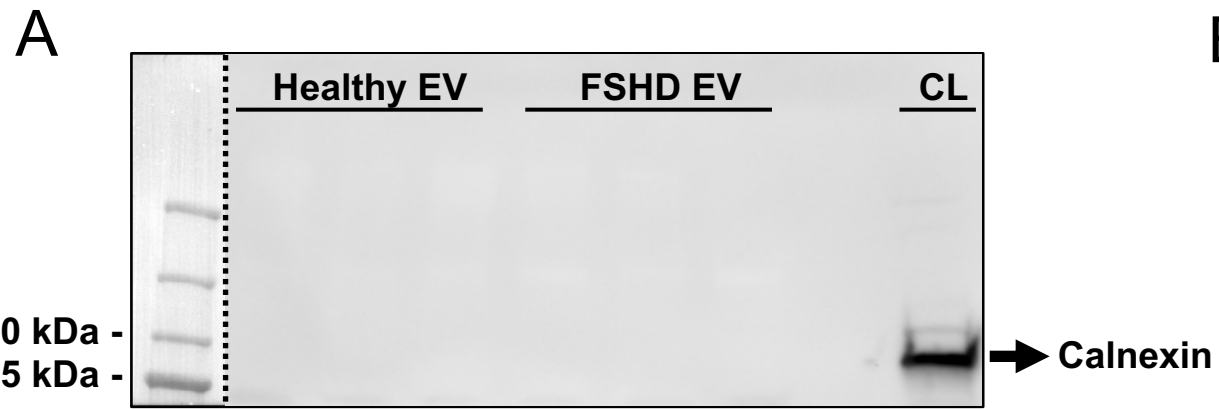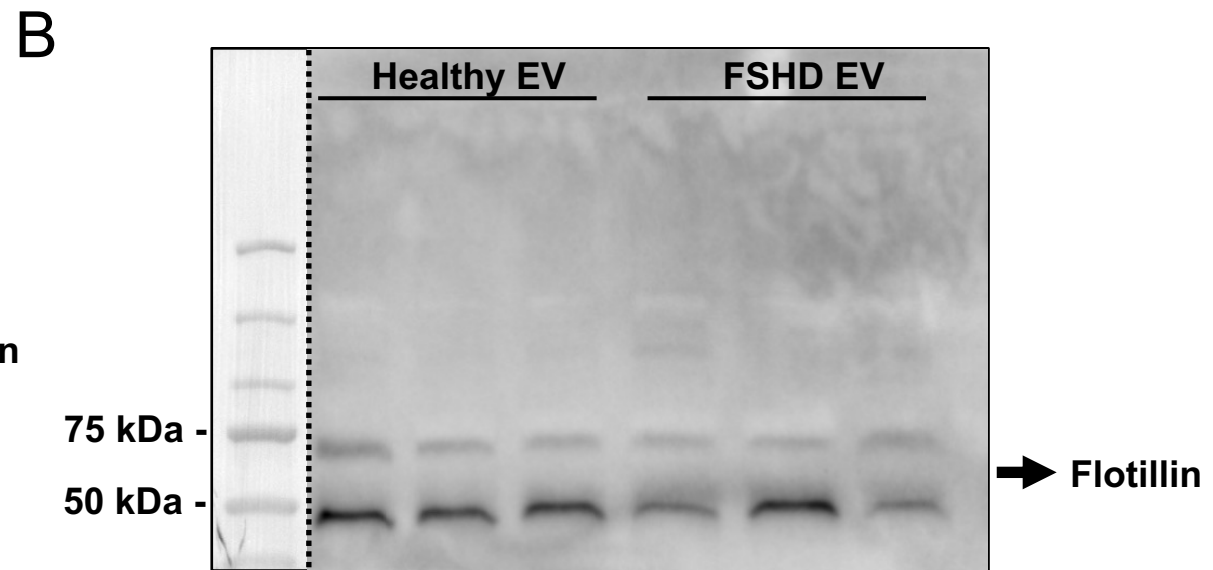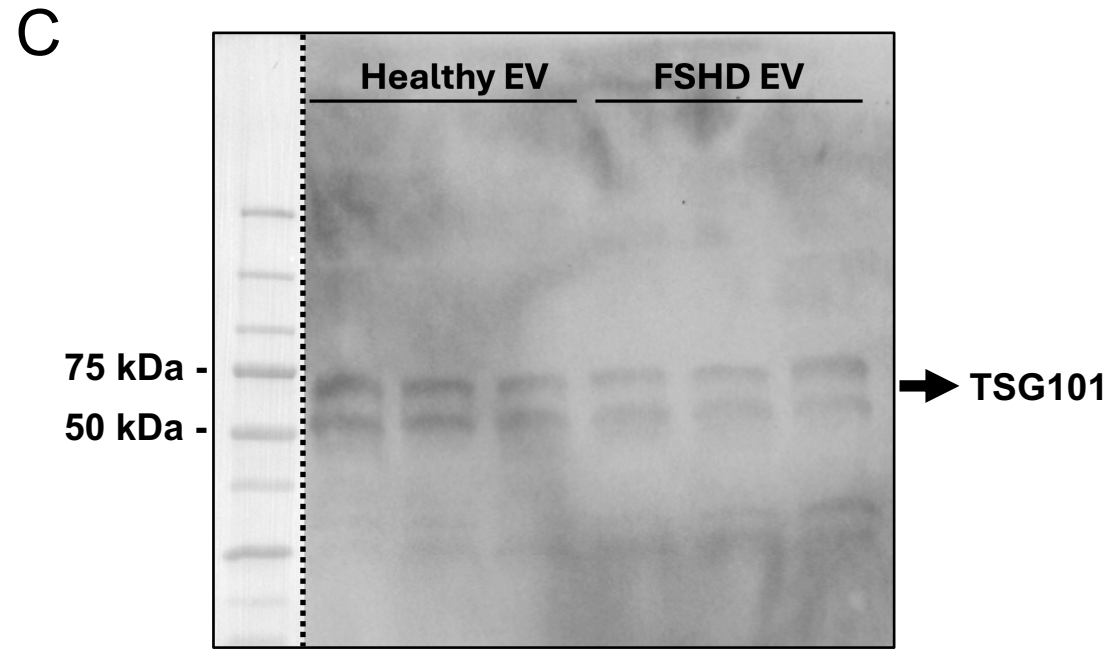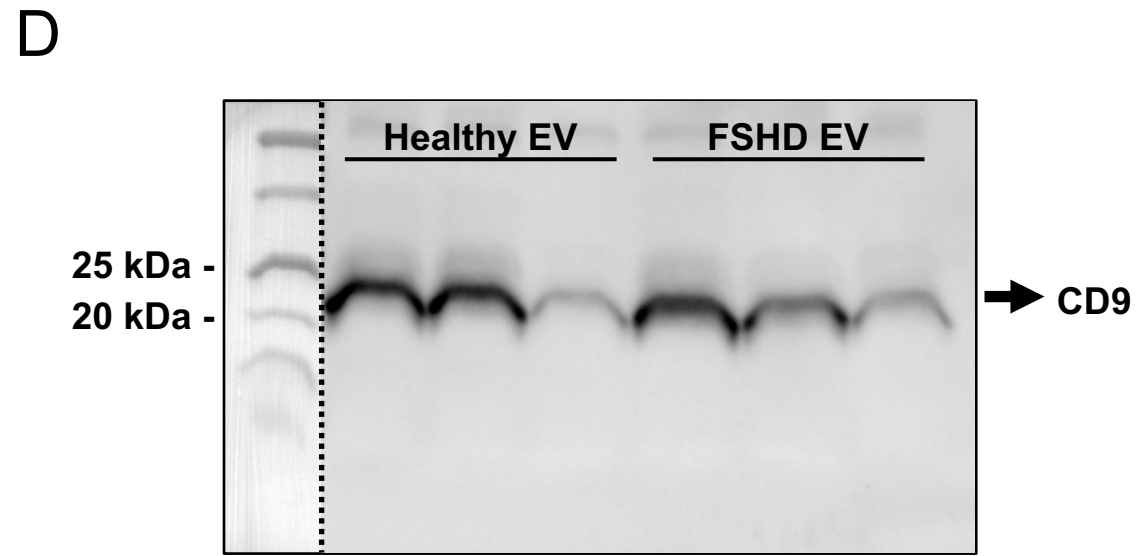

Supplement: Supplementary file 5 — Figure S5: Plasma EV identity confirmed by Western Blot. (A) Uncropped Western blot for Calnexin. (B) Uncropped Western blot for Flotillin. (C) Uncropped Western blot for TSG101. (D) Uncropped Western blot for CD9. CL: HEK293 cell lysis. [file ACN3-9999-0-s001.pdf]

1 h incubation

PNGase F

4 °C

50 °C

50 °C

75 kDa

50 kDa

38 kDa

LUM  
70 kDa

LUM  
38 kDa

Total

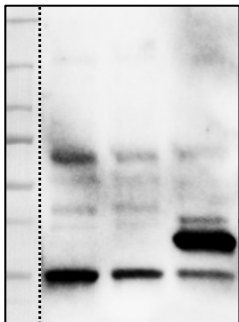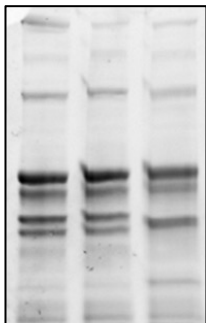

Supplement: Supplementary file 6 — Figure S6: Treatment of plasma EVs with PNGase F reduced the observed molecular weight of LUM. 25 μg of EV proteins were incubated with (i) PBS at 4°C for one‐hour (EV control), (ii) PBS at 50°C for one‐hour (heat control) (iii) PNGase F mix at 50°C for one‐hour. Stain‐free SDS‐PAGE gel was used to visualize total protein levels. [file ACN3-9999-0-s002.pdf]

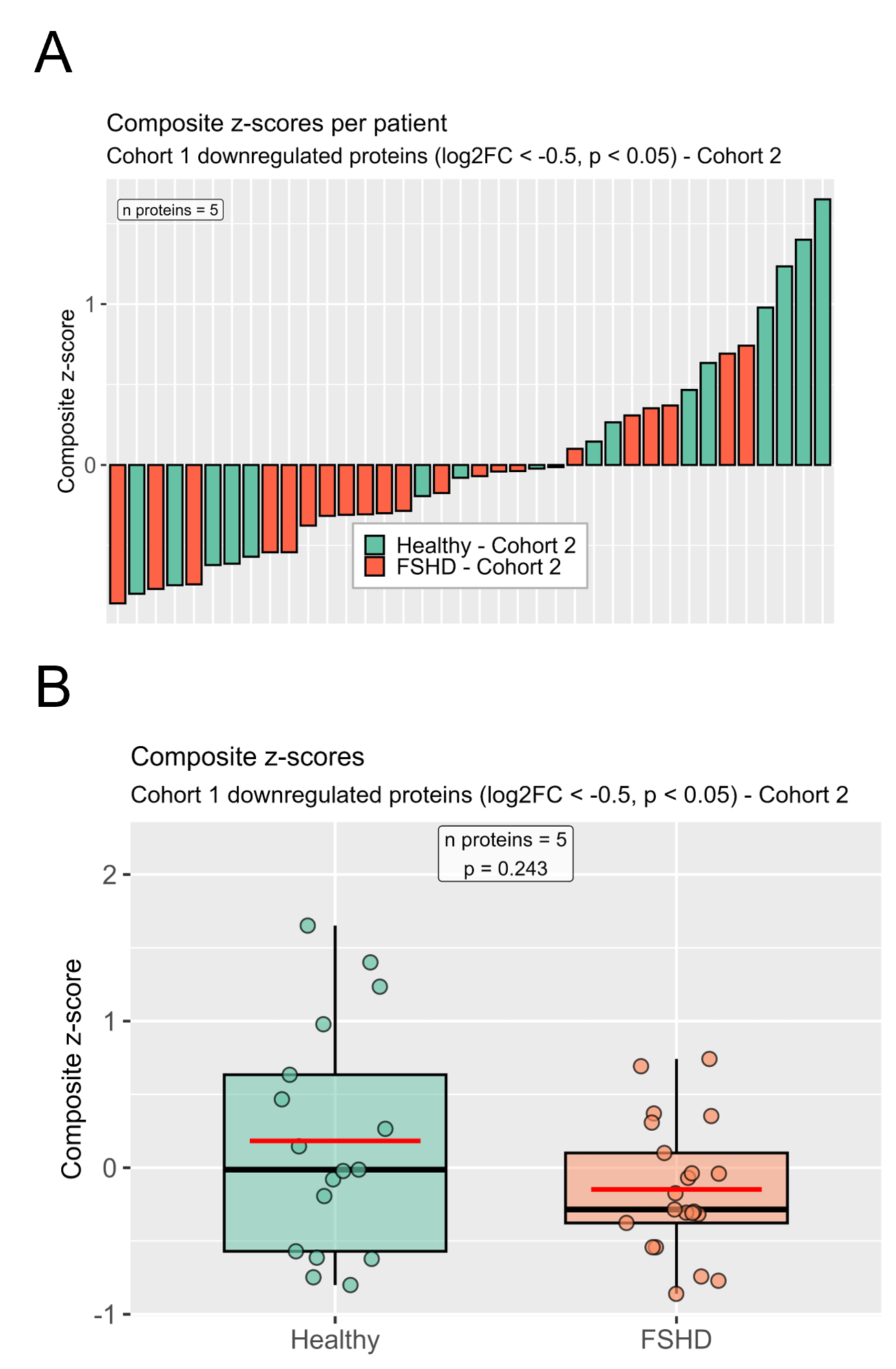

Supplement: Supplementary file 7 — Figure S7: Cross‐cohort evaluation of Cohort 1 downregulated protein signature in Cohort 2. (A) Composite z‐scores of a panel of five downregulated proteins (log2FC > −0.5, p < 0.05 in Cohort 1) are calculated and evaluated in FSHD1 patients and healthy controls of Cohort 2. All participants of Cohort 2 are ranked based on composite z‐scores for these select proteins. (B) Boxplots of composite z‐scores from (A). Points represent individual samples; boxes show interquartile range with median; red lines indicate means. p calculated using the Wilcoxon rank‐sum test. [file ACN3-9999-0-s014.tif]

**A**      Uncropped images relating to Fig. 3B

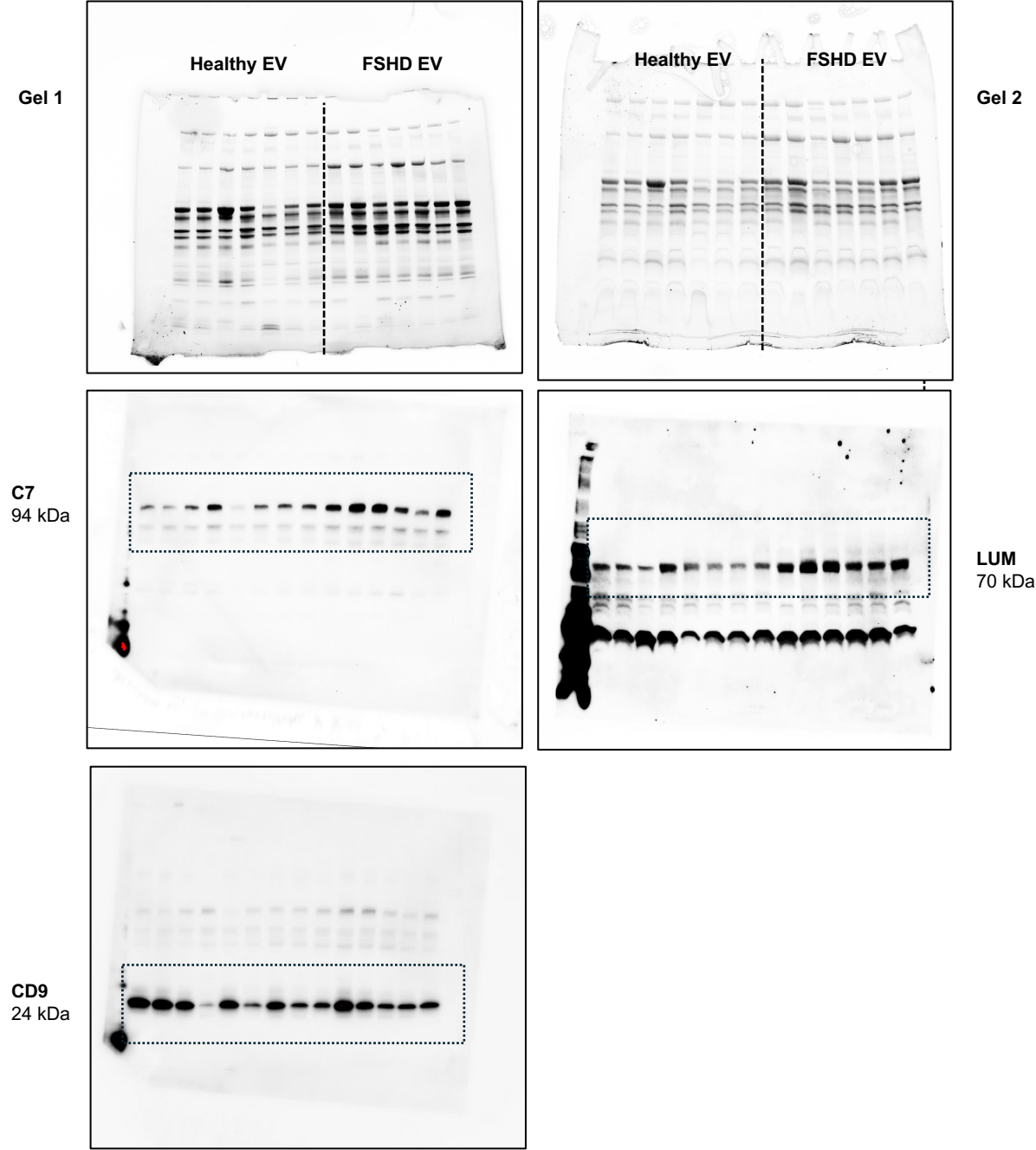

**B**      Uncropped images relating to Fig. 3E

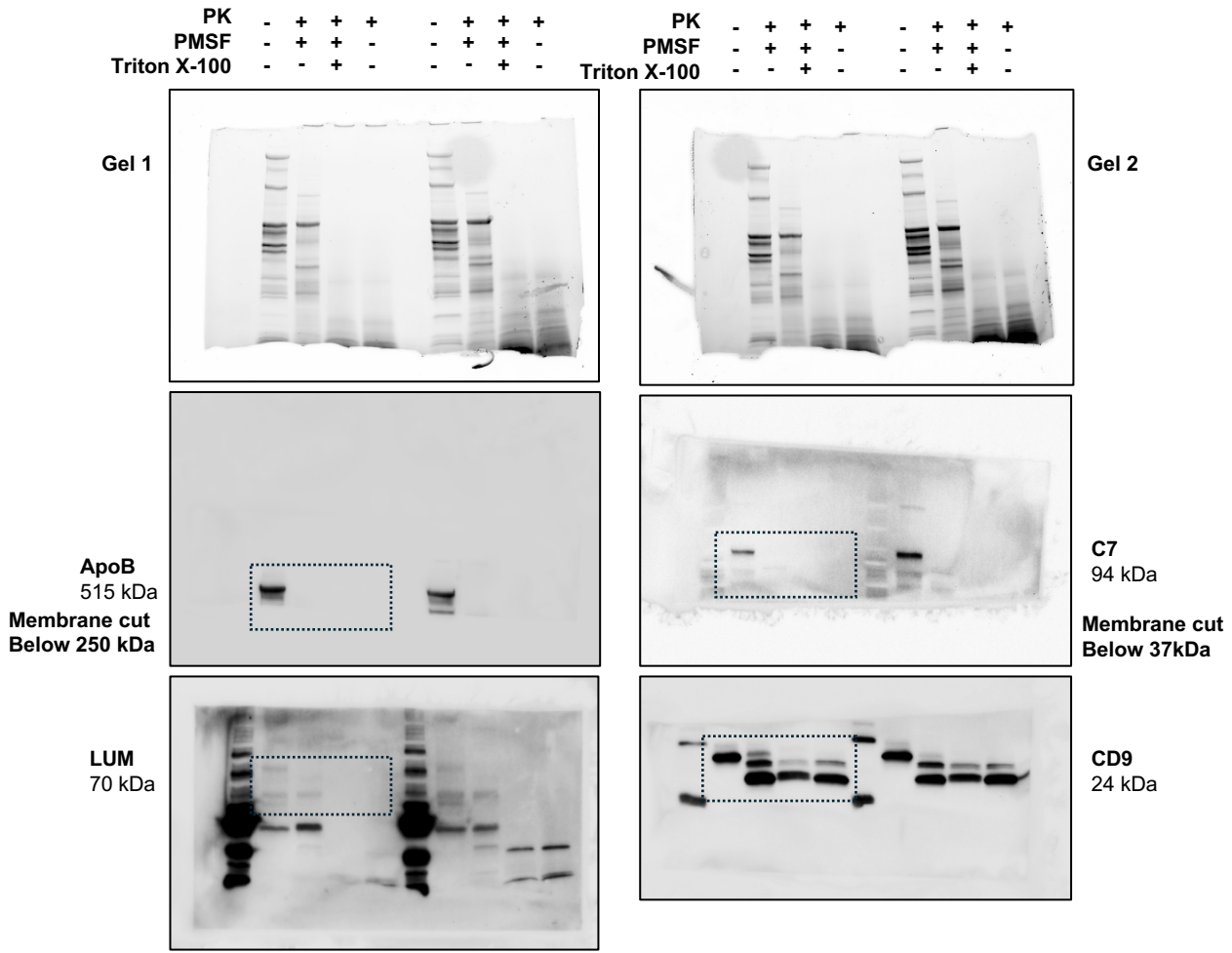

Supplement: Supplementary file 8 — Figure S8: Uncropped Western blot images. (A) Uncropped images of Western blots shown in Figure 3B. Cropped areas are indicated. (B) Uncropped images of Western blots shown in Figure 3E. Samples run in duplicates. Cropped areas are indicated. [file ACN3-9999-0-s003.pdf]
