# Supplementary material for: Plasma EV Proteomics Identifies ECM Remodeling and Inflammatory Proteins LUM and C7 as Candidate Biomarkers in FSHD
Source: Ann Clin Transl Neurol. 2026 May 20:10.1002/acn3.70435. Online ahead of print. doi: 10.1002/acn3.70435 (PMC13394447; doi:10.1002/acn3.70435)
Supplement: Supplementary file 12 — Table S4: Differentially abundant proteins (p < 0.05) in Cohort 1. Base Mean: Mean spectral counts in the healthy group. FC: Fold Change. Standard Error, p and FDR calculated with Wald Test. Age and sex were introduced as covariates. [file ACN3-9999-0-s008.docx]

| **Protein** | **Base Mean** | **FC in FSHD1** | **Std. Error** | ***p*** | **FDR** |
| --- | --- | --- | --- | --- | --- |
| C4BPB  FGG  CLU  PRG4  GPLD1  F13B  ITIH2  CNDP1  HPR  APOC2  COMP  MBL2  LAMB1 | 13.954  624.622  48.128  27.999  6.192  25.973  79.592  2.960  204.440  4.580  16.429  16.180  1.884 | 0.638  1.191  0.782  0.739  0.614  1.232  0.871  0.488  0.796  0.604  1.310  2.291  0.403 | 0.196  0.082  0.123  0.162  0.287  0.137  0.091  0.479  0.153  0.341  0.184  0.584  0.643 | 0.0009  0.0021  0.0037  0.0072  0.0139  0.0276  0.0290  0.0305  0.0314  0.0328  0.0343  0.0407  0.0414 | 0.1851  0.2132  0.2495  0.3607  0.5573  0.5868  0.5868  0.5868  0.5868  0.5868  0.5868  0.5868  0.5868 |
