# Supplementary material for: Plasma EV Proteomics Identifies ECM Remodeling and Inflammatory Proteins LUM and C7 as Candidate Biomarkers in FSHD
Source: Ann Clin Transl Neurol. 2026 May 20:10.1002/acn3.70435. Online ahead of print. doi: 10.1002/acn3.70435 (PMC13394447; doi:10.1002/acn3.70435)
Supplement: Supplementary file 13 — Table S5: Differentially abundant proteins (p < 0.05) in Cohort 2. Base Mean: Mean spectral counts in the healthy group. FC: Fold Change. Standard Error, p and FDR calculated with Wald Test. Age and sex were introduced as covariates. [file ACN3-9999-0-s011.docx]

| **Protein** | **Base Mean** | **FC in FSHD1** | **Std. Error** | ***p*** | **FDR** |
| --- | --- | --- | --- | --- | --- |
| TNC  ANPEP  C7  SVEP1  CFB  LUM  C9  CNDP1  FBLN1  APOA2  APOD  SERPINA1  C8G  THBS4 | 9.965  2.170  6.157  7.198  15.712  2.850  8.417  2.096  7.394  26.202  43.338  66.320  2.676  7.153 | 2.005  7.398  1.784  2.132  1.465  1.924  1.435  0.358  1.413  0.829  0.816  1.202  1.593  1.354 | 0.265  0.784  0.257  0.356  0.180  0.363  0.206  0.629  0.217  0.121  0.139  0.129  0.329  0.218 | 0.0002  0.0002  0.0012  0.0021  0.0022  0.0093  0.0115  0.0187  0.0213  0.0262  0.0346  0.0390  0.0414  0.0448 | 0.0268  0.0268  0.0901  0.1037  0.1037  0.3626  0.3812  0.5447  0.5523  0.6098  0.7338  0.7416  0.7416  0.7460 |
